# Nanostructured Fe-Doped Ni_3_S_2_ Electrocatalyst for the Oxygen Evolution Reaction with High Stability at an Industrially-Relevant Current Density

**DOI:** 10.1021/acsami.4c09821

**Published:** 2024-10-15

**Authors:** Jiahui Zhu, Wei Chen, Stefano Poli, Tao Jiang, Dominic Gerlach, João R. C. Junqueira, Marc C. A. Stuart, Vasileios Kyriakou, Marta Costa Figueiredo, Petra Rudolf, Matteo Miola, Dulce M. Morales, Paolo P. Pescarmona

**Affiliations:** † Chemical Engineering Group Engineering and Technology Institute Groningen (ENTEG), University of Groningen, 9747 AGGroningen The Netherlands; ‡ Department of Chemical Engineering and Chemistry, Eindhoven University of Technology, 5600 MBEindhoven, The Netherlands; § Zernike Institute for Advanced Materials, University of Groningen, 9747 AGGroningen, The Netherlands; ∥ Analytical Chemistry - Center for Electrochemical Sciences (CES), Faculty of Chemistry and Biochemistry, Ruhr University Bochum, D-44780Bochum Germany; ⊥ Electron Microscopy Group, Groningen Biomolecular Sciences and Biotechnology Institute, University of Groningen,9747 AG Groningen, The Netherlands

**Keywords:** oxygen evolution reaction, electrocatalyst, nanostructuring, Ni_3_S_2_, Fe doping

## Abstract

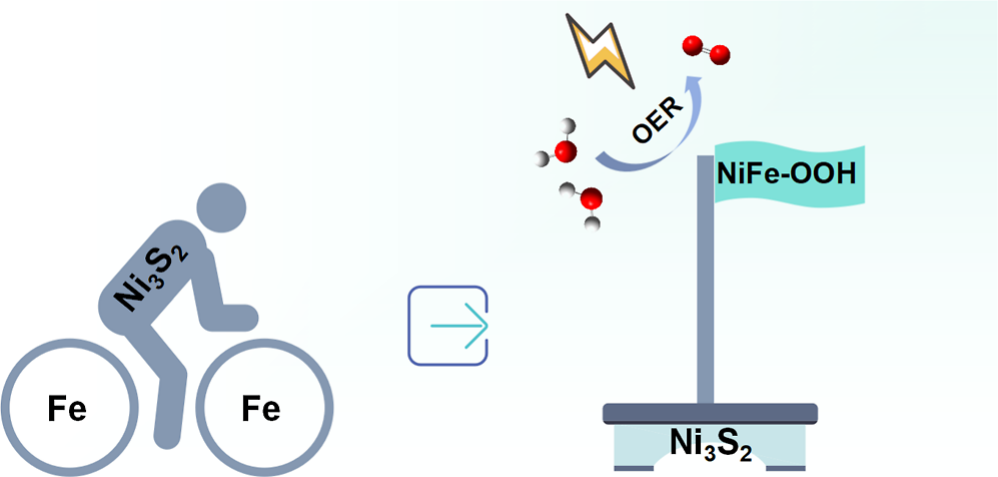

A novel oxygen evolution reaction (OER) electrocatalyst was prepared by a synthesis strategy consisting of the solvothermal growth of Ni_3_S_2_ nanostructures on Ni foam, followed by hydrothermal incorporation of Fe species (Fe–Ni_3_S_2_/Ni foam). This electrocatalyst displayed a low OER overpotential of 230 mV at 100 mA·cm^–2^, a low Tafel slope of 43 mV·dec^–1^, and constant performance at an industrially relevant current density (500 mA·cm^–2^) over 100 h in a 1.0 M KOH electrolyte, despite a minor loss of Fe in the process. Based on a detailed characterization by (in situ) Raman spectroscopy, (quasi-in situ) XPS, SEM, TEM, XRD, ICP-AES, EIS, and *C*_dl_ analysis, the high OER activity and stability of Fe–Ni_3_S_2_/Ni foam were attributed to the nanostructuring of the surface in the form of stable nanosheets and to the combination of Ni_3_S_2_ granting suitable electrical conductivity with newly formed NiFe-based (oxy)hydroxides at the surface of the material providing the active sites for OER.

## 1 Introduction

Electrochemical water splitting (EWS) is increasingly considered as a crucial sustainable technology to enable the production of green hydrogen (H_2_) from water (H_2_O), when powered by renewable sources such as solar or wind energy.^1−3^ Currently, alkaline water electrolysis (AWE) and proton-exchange membrane water electrolysis (PEMWE) are the main commercially available low-temperature water electrolysis technologies.^4,5^ AWE, as opposed to PEMWE, can avoid the need for costly noble metal electrocatalysts. In addition, some of the drawbacks of AWE, such as gas crossover and alkaline corrosion, may be minimized by using the anion-exchange membrane water electrolysis (AEMWE) technology, which ideally combines the advantages of AWE with the cell configuration of PEMWE.^6−8^ Nonetheless, the high electric power consumption remains a key issue that limits the widespread adoption of AWE. The electric power demands depend largely on the overpotential of the hydrogen evolution reaction (HER, two-electron-transfer cathodic reaction, 2H_2_O + 2 e^–^ → H_2_ + 2OH^–^) and, particularly, of the oxygen evolution reaction (OER, four-electron-transfer anodic reaction, 4 OH^–^ → 2H_2_O + O_2_ + 4 e^–^).^9,10^ Typically, Ni-based materials (such as stainless steel, Raney Ni, and Ni alloys) are utilized as OER catalysts in industrial AWE as they are cost-effective, though they suffer from the above-mentioned high overpotentials.^11−13^ Therefore, several approaches have been investigated to boost the OER activity of Ni-based electrodes.^14−20^ These strategies typically rely on increasing the specific surface area of the electrode, for example by enhancing its roughness,^21−23^ or increasing the activity of the surface species, for example by including additional elements such as Fe or Co.^24,25^ Among these options, the decoration of Ni-based electrodes with transition metal sulfides, especially nickel sulfides, has attracted widespread attention due to the increase in electrocatalytic activity that they bring about, combined with the low cost and the high abundance of the substances used for their synthesis.^26−28^ Different hypotheses have been put forward to explain the enhanced electrocatalytic performance obtained with Ni sulfides compared to Ni-based electrocatalysts prepared without sulfur.^12,29,30^ Studies based on Raman spectroscopy indicate that the surface of sulfides spontaneously undergoes reconstruction under OER operating conditions to form amorphous (oxy)hydroxides.^31−33^ For example, Xu et al. reported that NiOOH species are generated on the surface of F–Ni_3_S_2_ during the OER process.^34^ Zhang et al. reported that γ-NiOOH and γ-FeOOH are formed according to in situ Raman spectroscopy of Fe–NiO/NiS_2_.^35^ Wang et al. reported that the Raman spectra of Fe-MOF-Ni_3_S_2_/NF indicate the presence of NiOOH after OER testing.^36^ The formation of these surface (oxy)hydroxides is of crucial importance because several studies have indicated that these are the electrocatalytically active species.^37−39^ The nanostructures that generally characterize the surface of (oxy)hydroxides generated from nickel sulfides are an additional asset of these electrocatalysts, as they grant a high specific surface area. However, it is important to restrict the formation of the amorphous (oxy)hydroxides to the surface of the material as these compounds exhibit poor electrical conductivity (estimated to be as low as ∼10^–15^ S·m^–1^),^40,41^ which hinders the overall electrocatalytic performance when these materials are used as electrocatalysts in their bulk form. In this context, an important asset of nickel sulfides, and especially of Ni_3_S_2_, is the relatively high electrical conductivity compared to nickel (oxy)hydroxides (σ_Ni3S2_ = 5.6 × 10^6^ S·m^–1^, which is several orders of magnitude higher than that of (oxy)hydroxides, though still lower than that of metallic Ni, σ_Ni_ = 1.4 × 10^7^ S·m^–1^).^42^ Therefore, the combination of a metal sulfide granting suitable electrical conductivity with a thin surface layer consisting of (oxy)hydroxides providing abundant active sites for the OER might prove beneficial for the overall performance, thus explaining the observed promising results obtained with nickel sulfides.

It should be noted that most Ni_3_S_2_-based electrocatalysts are prepared in the form of powders and, therefore, need to be mixed with polymer binders and/or organic solvents to form inks, which are then deposited on conductive substrates. However, the use of polymer binders may compromise the exposure of active sites, while non-optimized catalyst inks and deposition methods may lead to the formation of agglomerates at the electrode surface, which can substantially increase the overall resistance. In the perspective of a large-scale application, it is preferable to prepare nanostructured Ni_3_S_2_ directly on conductive substrates. Ni foam, with its outstanding conductivity and three-dimensional porous structure, has emerged as a viable conductive support. Also, Ni foam can serve as a source of nickel for preparing Ni_3_S_2_. Moreover, previous investigations have demonstrated that doping with different elements can promote the electrocatalytic activity of Ni_3_S_2_.^43−45^ More specifically, it has been shown that Fe plays a critical role in enhancing the OER activity of Ni_3_S_2_-based catalysts. For instance, Zhang et al. reported that the catalytic activity of Ni_3_S_2_ is boosted by the incorporation of iron, and the prepared Fe–Ni_3_S_2_ exhibited a superior activity compared to NiFe-layered double hydroxide (LDH).^12^ Cheng et al. synthesized a Fe-doped Ni_3_S_2_ particle film with a low overpotential of 253 mV at a high current density of 100 mA·cm^–2^.^46^ Bao et al. reported that Fe^II^ dopants enhance the OER performance of Ni_3_S_2_ and attributed this to the changes in electron density caused by doping.^47^ All of these studies indicate that constructing nanostructures and doping with Fe are effective strategies to enhance the performance of the OER of Ni_3_S_2_-based electrocatalysts.

Motivated by these considerations, we aimed to synthesize an improved OER electrocatalyst by developing a new synthesis strategy consisting of the solvothermal growth of Ni_3_S_2_ nanostructures on Ni foam through treatment with elemental S, followed by hydrothermal doping with Fe (Fe–Ni_3_S_2_/Ni foam). Our approach provides a straightforward and potentially up-scalable way of preparing a highly active OER electrocatalyst that utilizes Ni foam as a source of nickel for preparing the sulfide. Importantly, we demonstrated that our Fe–Ni_3_S_2_/Ni foam electrocatalyst enables carrying out the OER at an industrially relevant current density (up to 500 mA·cm^–2^) with low overpotential (0.42 V, corresponding to an applied potential of 1.65 V vs RHE), displaying a stable performance under these operating conditions for 100 h. Furthermore, the nature of the OER active sites of Fe–Ni_3_S_2_/Ni foam was investigated by in situ Raman spectroscopy and X-ray photoelectron spectroscopy (XPS) under different electrode potentials.

## 2 Experimental Section

### 2.1 Electrocatalyst Synthesis

#### 2.1.1 Chemicals and Materials

Ni foam (99.5% purity, 0.25 mm thickness) was purchased from Xiamen Tmax Battery Equipment Limited, China. Ni mesh (58.69 g·mol^–1^, 0.1 mm thickness) was purchased from Alfa Aesar. Sulfur powder (S, ≥99.0%), sodium borohydride (NaBH_4_, powder, ≥98.0%), iron(II) sulfate heptahydrate (FeSO_4_·7H_2_O, ACS reagent, ≥99.0%), potassium hydroxide (KOH, semiconductor grade, pellets, 99.99%), and hydrochloric acid (37% aqueous HCl solution, ACS reagent) were purchased from Sigma-Aldrich. Absolute ethanol (EtOH, ≥99.5%) was purchased from J.T. Baker. Ethanol (EtOH, 96%) was purchased from Boom B.V. All chemicals were used without any further purification. Water of Milli-Q grade (18.25 M Ω·cm) was used in this work.

#### 2.1.2 Pretreatment of the Ni Foam

The commercial Ni foam was cut from its original A4 format to the desired size (2.3 cm × 2.3 cm) using scissors (the deviation between different plates was <0.05 cm). Each plate was first sonicated with 6 M HCl solution, then with ethanol, and finally with Milli-Q water for 15 min each, to clean the surface of the Ni foam, and then dried for use.

#### 2.1.3 Synthesis of Ni_3_S_2_/Ni Foam

Ni_3_S_2_ nanothreads supported on Ni foam (Ni_3_S_2_/Ni foam) were prepared by adapting a method reported previously.^48^ In detail, 1 mmol of S powder and 1.5 mmol of NaBH_4_ were weighed in a glass beaker and then dissolved in 15 mL of absolute ethanol. The obtained yellow, transparent solution was stirred with a magnetic stirrer for 10–15 min at room temperature until it turned milky white (indicative of NaHS formation). Next, this sample was transferred into a 25 mL Teflon-lined stainless steel autoclave (TEFIC, China). Then, a Ni foam (pretreated as described above) was placed obliquely into the autoclave, so that it was completely below the liquid level. The autoclave was then closed, placed in an oven, and maintained statically at 160 °C for 12 h. Finally, the modified Ni foam was collected, washed with ethanol and Milli-Q water, and then dried at 80 °C for 12 h. The obtained sample was labeled Ni_3_S_2_/Ni foam.

Note: both the original and the modified Ni foam plates were always handled using only Teflon tweezers.

#### 2.1.4 Synthesis of Fe–Ni_3_S_2_/Ni foam

Nanostructured Fe-doped nickel sulfide on Ni foam (Fe–Ni_3_S_2_/Ni foam) was synthesized by a hydrothermal reaction. Specifically, 15 mL of a clear aqueous solution containing 0.1 mmol FeSO_4_·7H_2_O was prepared and then placed into a 25 mL Teflon-lined autoclave together with Ni_3_S_2_/Ni foam (placed obliquely). The autoclave was then closed, placed in an oven, and kept statically at 80 °C for 1 h. A reference material was prepared with the method just described but replacing Ni_3_S_2_/Ni foam with Ni foam to prepare Fe-based (oxy)hydroxides on Ni foam (Fe–Ni foam).

### 2.2 Physicochemical Characterization

#### 2.2.1 Characterization of the Prepared Electrocatalysts

A Bruker D-8 Advance Spectrometer was used to record the X-ray diffraction (XRD) patterns employing a 0.25° divergent slit and a 0.125° antiscattering slit. The patterns were recorded in the 2θ range from 20 to 80°, in steps of 0.02°, and a counting time of 2 s per step. The X-ray beam was generated by Cu Kα radiation with λ = 1.5418 Å. Raman spectra were recorded with excitation at λ = 785 nm using an Olympus BX51 microscope equipped with a fiber-coupled laser (BT785, ONDAX), a fiber-coupled Shamrock163i spectrograph, and an iVac-324FI CCD camera. The laser power with which the sample was irradiated was 2 mW. Spectra were acquired with an Andor SOLIS software and processed with Spectragryph-On (F. Menges, version 1.2.14). Each spectrum was the sum of 30 scans, with a collection time of 5 s each. Inductively coupled plasma–atomic emission spectroscopy (ICP–AES) measurements were performed on an Optima 7000 DV ICP–OES Spectrometer. All solid samples were prepared through acid digestion. A small amount of sample (around 10 mg) was weighed. Then, 7 mL of aqueous HNO_3_ (68 wt %) was added, and microwave-assisted digestion was performed with the following temperature program: heating up to 200 °C within 10 min and then 10 min at 200 °C. Once sample dissolution was achieved, 50 mL of double-distilled water was added. For the liquid solutions (i.e., the KOH aqueous solutions), the sample was diluted and measured directly. Scanning electron microscopy (SEM) was performed on an FEI Nova Nano SEM 650 operated at 18 kV with a spot size of 3.5 nm. Transmission electron microscopy (TEM) was carried out on a FEI Tecnai T20 at 200 keV. Prior to TEM analysis, the synthesized samples were cut into ca. 2 mm × 2 mm pieces and sonicated in 2 mL of ethanol for 2–5 min. Next, one drop of the ethanol suspension containing the fragments removed by sonication from the Ni foam was placed on a carbon-coated TEM 400 mesh copper grid. The same microscope, operating in the scanning mode (STEM) and equipped with a silicon drift detector (Xmax 80T, Oxford Instruments), was used for elemental analysis by energy-dispersive X-ray spectroscopy (EDX). X-ray photoelectron spectroscopy (XPS) measurements were performed using a Surface Science Instruments SSX-100 ESCA spectrometer equipped with a monochromatic aluminum anode (Kα = 1486.6 eV). The pressure in the measurement chamber was maintained below 8 × 10^–9^ mbar during data acquisition. The electron take-off angle with respect to the surface normal was 37°. The diameter of the analyzed area was 1000 μm; the energy resolution was 1.26 eV (or 1.67 eV for a broad survey scan). The XPS spectra were analyzed using the least-squares curve fitting program Winspec developed at the LISE, University of Namur, Belgium, and included a Shirley baseline subtraction and fitting with a minimum number of peaks consistent with the expected composition of the probed volume, taking into account the experimental resolution. Peak profiles were taken as a convolution of Gaussian and Lorentzian functions. Binding energies were referenced to the C 1s photoemission peak originating from adventitious carbon (C–C/C=C), which was set at a binding energy of 284.8 eV. All binding energies derived from deconvolution have an uncertainty of ±0.1 eV. All measurements were carried out on freshly prepared samples, and three different spots were measured on each sample to check for homogeneity.

#### 2.2.2 In situ Raman Spectroscopy

In situ Raman spectroscopy was performed with a Lab-RAM HR Raman microscopy system (Horiba Jobin Yvon, HR550) equipped with a 532 nm laser as the excitation source, a water immersion objective (Olympus LUMFL, 60×, numerical aperture = 1.10), a monochromator (1800 grooves·mm^–1^ grating), and a Synapse CCD detector. The laser power was 1.5 mW. Each spectrum is the sum of 4 scans with a collection time of 60 s each. A three-electrode electrochemical cell was used for these in situ Raman spectroscopy tests. A Pt mesh and Ag | AgCl | KCl (3 M) were used as counter and reference electrodes, respectively. To protect the objective from corrosion, 0.01 M KOH was used instead of the conventional 1.0 M KOH solution. In situ Raman analysis combined with electrochemical characterization was carried out simultaneously at selected potentials vs RHE to obtain the surface chemical composition and structural information of the materials. The collection of the Raman spectra at each potential started 30 s after that potential was initially applied. In particular, the Raman spectra of Ni_3_S_2_/Ni foam and Fe–Ni_3_S_2_/Ni foam were first collected after 20 min at 0.99 V vs Ag | AgCl | KCl (∼1.9 V vs RHE) and then reacquired after 30 s at 0.29 V vs Ag | AgCl | KCl (∼1.2 V vs RHE).

#### 2.2.3 Quasi-in situ X-ray Photoelectron Spectroscopy

Quasi-in situ XPS was conducted on a SPECS Phoibos NAP-150 electron analyzer with a monochromatic Al Kα X-ray source (*h*ν = 1486.6 eV). The pressure in the measurement chamber was maintained below 8 × 10^–9^ mbar during data acquisition. The electron take-off angle with respect to the surface normal was 37°. The diameter of the analyzed area was 1000 μm; the energy resolution was 1.26 eV (or 1.67 eV for a broad survey scan). To prepare the electrode, the Ni_3_S_2_/Ni foam or Fe–Ni_3_S_2_/Ni foam was first cut into small pieces and then placed in an ethanol solution and sonicated for 6 h. In this way, a suspension containing fragments from the surface of our electrocatalyst was obtained. Afterward, Vulcan XC 72 carbon and Sustainion ionomer (1H-imidazole, 1,2,4,5-tetramethyl-, compd. with 1-(chloromethyl)-4-ethenylbenzene polymer with diethynylbenzene and ethenylbenzene, and 1,2,4,5-tetramethylimidazole) were added to the suspension. Vulcan XC 72 carbon powder was included in the formulation to improve the electrical conductivity, while the Sustainion ionomer was used as a binder. It is worth noting that the XPS signal of Vulcan XC 72 did not show observable amounts of Ni and Fe (Figure S2). Finally, the suspension was coated with glassy carbon. A three-electrode electrochemical cell was used for these quasi-in situ XPS tests. A Pt wire and Hg | HgO | KOH (1 M) were used as counter and reference electrodes, respectively. 1.0 M KOH aqueous solution was used as the electrolyte. The quasi-in situ XPS analysis started with electrochemical reaction at a selected potential for 10 min in a gas-tight chamber purged with N_2_. After the reaction, the electrolyte was removed, and the working electrode was transferred to the ultrahigh vacuum (UHV) chamber for XPS testing without air contact (in a gas-tight chamber purged with inert gas). The XPS data at different potentials were obtained from different electrodes from the same synthesis batch of Ni_3_S_2_/Ni foam or Fe–Ni_3_S_2_/Ni foam. The O 1s photoemission peak from M–OH was used as a reference for the binding energies, and it was set at 531 eV. All binding energies derived from deconvolution have an uncertainty of ±0.1 eV.

### 2.3 Electrochemical Measurements

#### 2.3.1 H-Type Electrolytic Cell

The OER performance of the as-synthesized electrocatalysts was investigated in a 1.0 M KOH aqueous solution (prepared with Milli-Q water) using an H-type double-chamber electrolytic cell connected to a potentiostat (Gamry Interface 1000, USA), with each chamber of the cell having a volume of 50 mL and being separated from each other by a glass filter (P1). All electrochemical tests in this section were carried out at 30 °C, controlled by a thermostat (Julabo MV-BASIS, Germany). The as-synthesized modified Ni foams were cut into a size of 0.5 × 1.5 cm, of which 0.5 × 0.6 cm were immersed in the electrolyte solution and thus used as the working electrode. The geometric area of the working electrode was 0.6 cm^2^ because both sides of the modified Ni foam are expected to contribute to the OER. Ni mesh (ca. 2 cm × 2 cm) and Hg | HgO | KOH (1 M) electrode were employed as the counter and reference electrodes, respectively.

All potentials in this section were converted and referenced to the reversible hydrogen electrode (RHE) by first measuring the potential difference between the Hg | HgO | KOH (1 M) electrode and the (RHE, Gaskatel) at 30 °C in 1.0 M KOH aqueous solution for 100 s, which gave a stable value of 0.915 V (Figure S1). This value was then used to convert the electrode potentials to the RHE scale according to the formula:
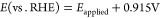
1

At a scan rate (ν) of 5 mV·s^–1^, linear sweep voltammetry (LSV) curves were recorded in the potential range of 0.3–1.0 V vs Hg | HgO | KOH. Based on the second LSV curve, the overpotential (η) and Tafel slope (*b*) of the electrode were calculated using the following two formulas:
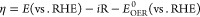
2
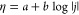
3where *i* [*A*] is the current, *R* [Ω] is the uncompensated resistance, as determined by electrochemical impedance spectroscopy (EIS) measured at the corresponding open-circuit potentials (from 100 to 1000 Hz) with 10 mV amplitude (root-mean-square) before measuring LSV, *E*^0^_OER_ (vs RHE) is 1.23 V, and *j* [mA·cm^–2^] is the current density calculated with respect to the geometric area.

EIS measurements were additionally performed from 0.01 to 100 kHz, at 1.53 V vs RHE (*η* = 300 mV) and 1.63 V vs RHE (*η* = 400 mV) with an amplitude of 10 mV (root-mean-square).

To determine the double-layer capacitance (*C*_dl_), cyclic voltammetry (CV) curves were acquired in the non-Faradaic potential region with various scan rates (25–200 mV·s^–1^).^49^ The measurement was done by using surface mode sampling. When the scan rates were 25, 50, or 100 mV·s^–1^, the step size was 2 mV, and the current range was fixed to 0.1 mA. When the scan rates were 150 and 200 mV·s^–1^, the step sizes were 3 and 4 mV, respectively, and the current range was fixed to 1 mA. *C*_dl_ was estimated according to the following formula:
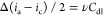
4in which *i*_a_ and *i*_c_ are the anodic and cathodic currents, respectively, and *ν* is the scan rate.

The *C*_dl_ [mF] was used to determine the effective electrochemically active surface area (ECSA) using the following formula:
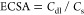
5where *C*_s_ [mF·cm^–2^] is the specific capacitance.

The stability of selected electrocatalysts was investigated by chronopotentiometric (CP) tests, which were carried out at a high current density of 500 mA·cm^–2^ for 100 h using a syringe pump (CBN NE-300, USA) to replenish the water gradually consumed (and possibly lost by evaporation) during the test. The flow rate of the syringe pump was calculated based on the following steps.

First, the number of electrons involved in the half-reaction was obtained according to the following formula:
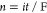
6in which *i* is the applied current, *t* [min] is the reaction time, F is the Faraday constant (96485.33C·mol^–1^), and *n* [mol] is the number of electrons.

Then, the theoretical volume of water was calculated based on the cathodic reaction (2H_2_O + 2 e^–^ → H_2_ + 2 OH^–^) of water electrolysis and the two following formulas:
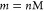
7
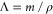
8where *m* [*g*] is the mass of water, M [g·mol^–1^] is the molar mass of water, Λ [μL] is the volume of water, and *ρ* [g·cm^–3^] is its density.

Finally, the flow rate was determined by the following formula:
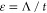
9where *ε* [μL·min^–1^] is the flow rate.

#### 2.3.2 Water Splitting Investigation in a 5 cm^2^ AEM Electrolyzer Cell

The overall water splitting performance of the prepared electrodes in a full cell setup was carried out by adapting a commercial AEM electrolyzer cell driven by an 8-channel IVIUM-*n*-Stat electrochemical workstation (module 10A/5 V^–1^ MHz). The commercial cell contains two Ni plates with channels through which the electrolyte flows on the inner side of the Ni plates (Figure 10a). Square-shaped 5 cm^2^ Ni foam and Fe–Ni_3_S_2_/Ni foam as the cathode (−) and anode (+), respectively, were assembled horizontally with the Sustainion X37–50 grade 60 membrane as a separator between the two Ni plates of the commercial AEM electrolyzer cell, using gaskets to prevent liquid leakage (Figure 10a). The Ni plates also serve as current collectors on each side of the cell. The cell was operated vertically, and a 1.0 M KOH aqueous electrolyte was pumped through the cell at a flow rate of 300 mL·min^–1^. After 30 min of pretreatment at a constant current of 0.5 A, the cell current as a function of the applied cell potential was recorded with a scan rate of 5 mV·s^–1^ and a maximum current of 5 A. CP measurements were performed at a current of 5 A for 100 h to explore the stability. As a reference, the AEM electrolyzer mounted with bare Ni foam as both the anode and the cathode was also tested under the same conditions.

**Figure 1:**
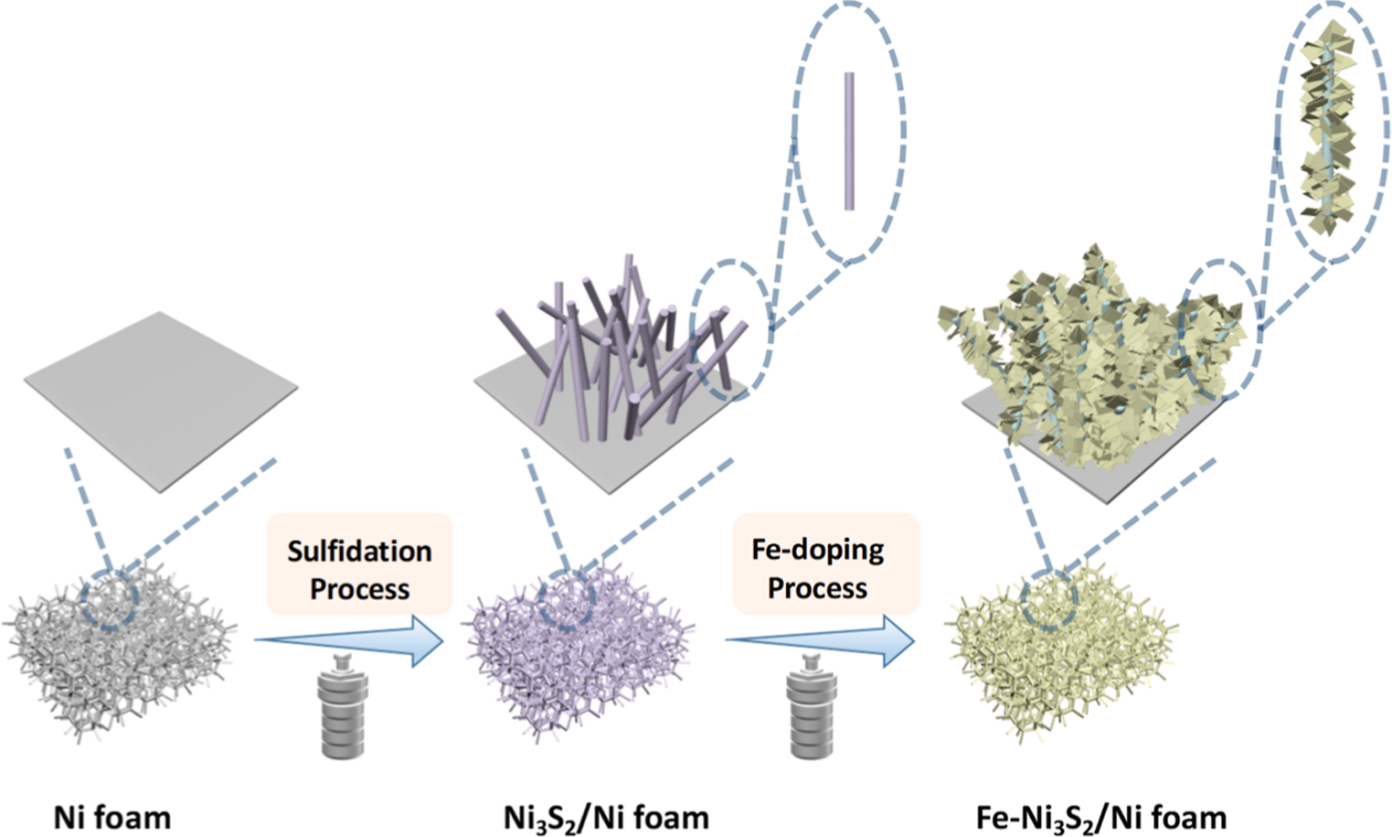
Schematic diagram of the synthesis of Fe–Ni_3_S_2_/Ni foam and of its precursor (Ni_3_S_2_/Ni foam).

**Figure 2:**
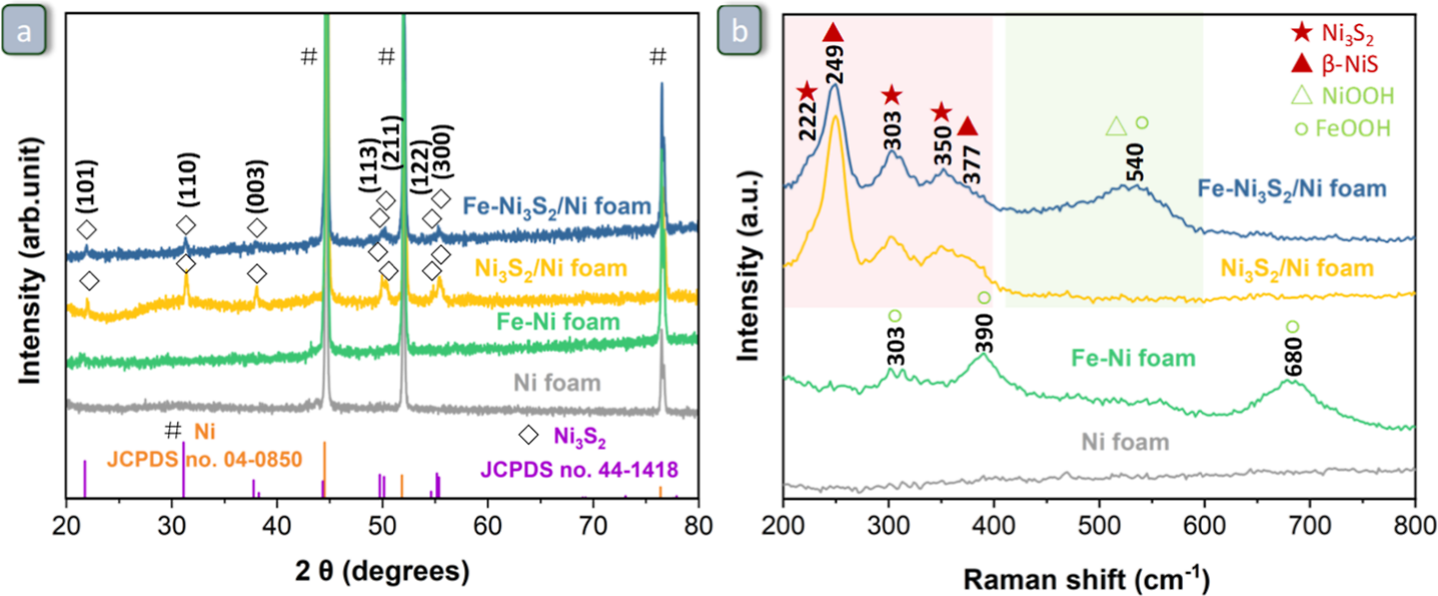
(a) XRD patterns and (b) Raman spectra of Ni foam, Fe–Ni foam, Ni_3_S_2_/Ni foam, and Fe–Ni_3_S_2_/Ni foam.

**Figure 3:**
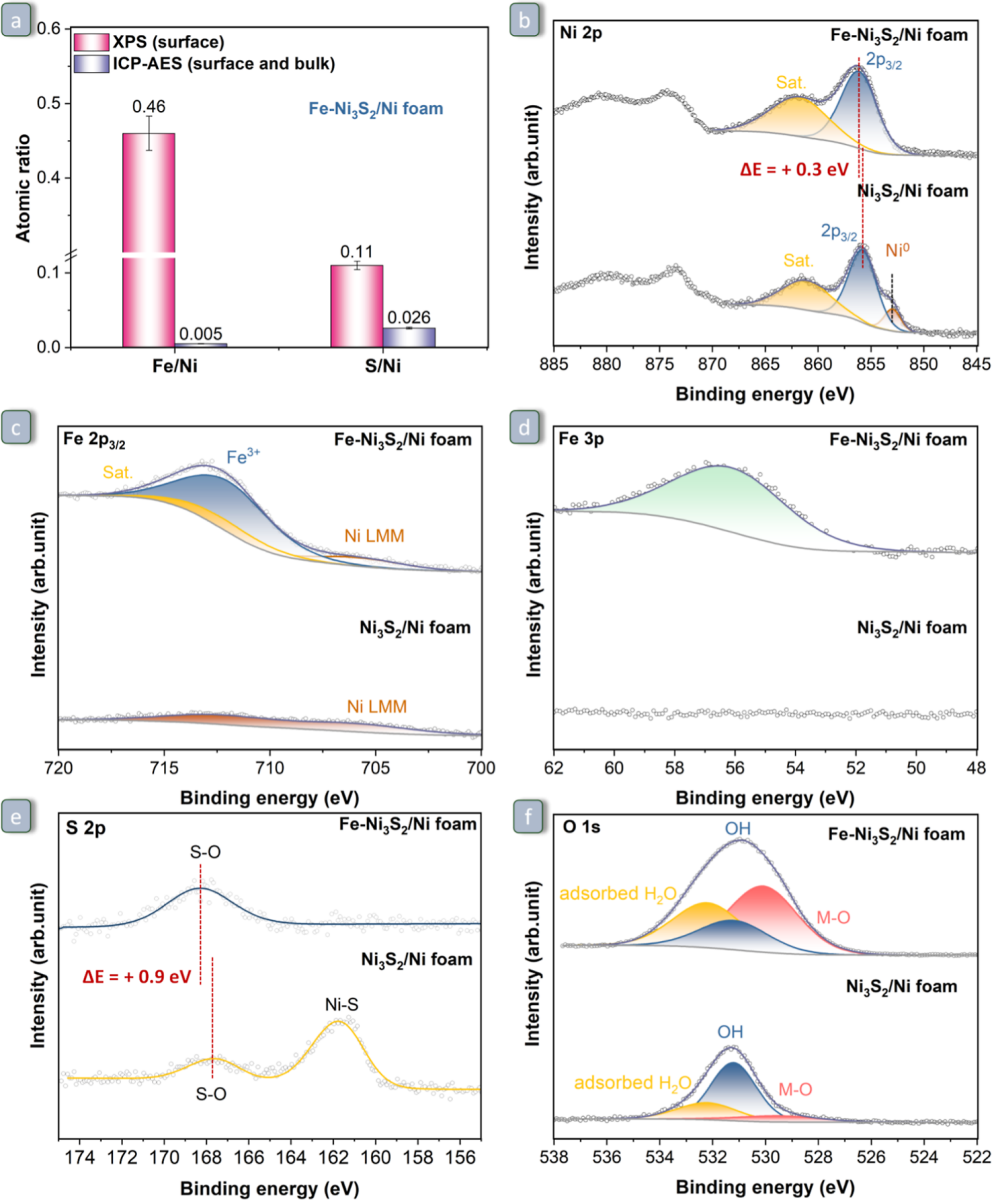
(a) Fe/Ni and S/Ni atomic ratios in Fe–Ni_3_S_2_/Ni foam at the surface (measured by XPS) and in the whole material (measured by ICP-AES). XPS signals of the (b) Ni 2p, (c) Fe 2p_3/2_, (d) Fe 3p, (e) S 2p, and (f) O 1s core level regions of Ni_3_S_2_/Ni foam and Fe–Ni_3_S_2_/Ni foam.

**Figure 4:**
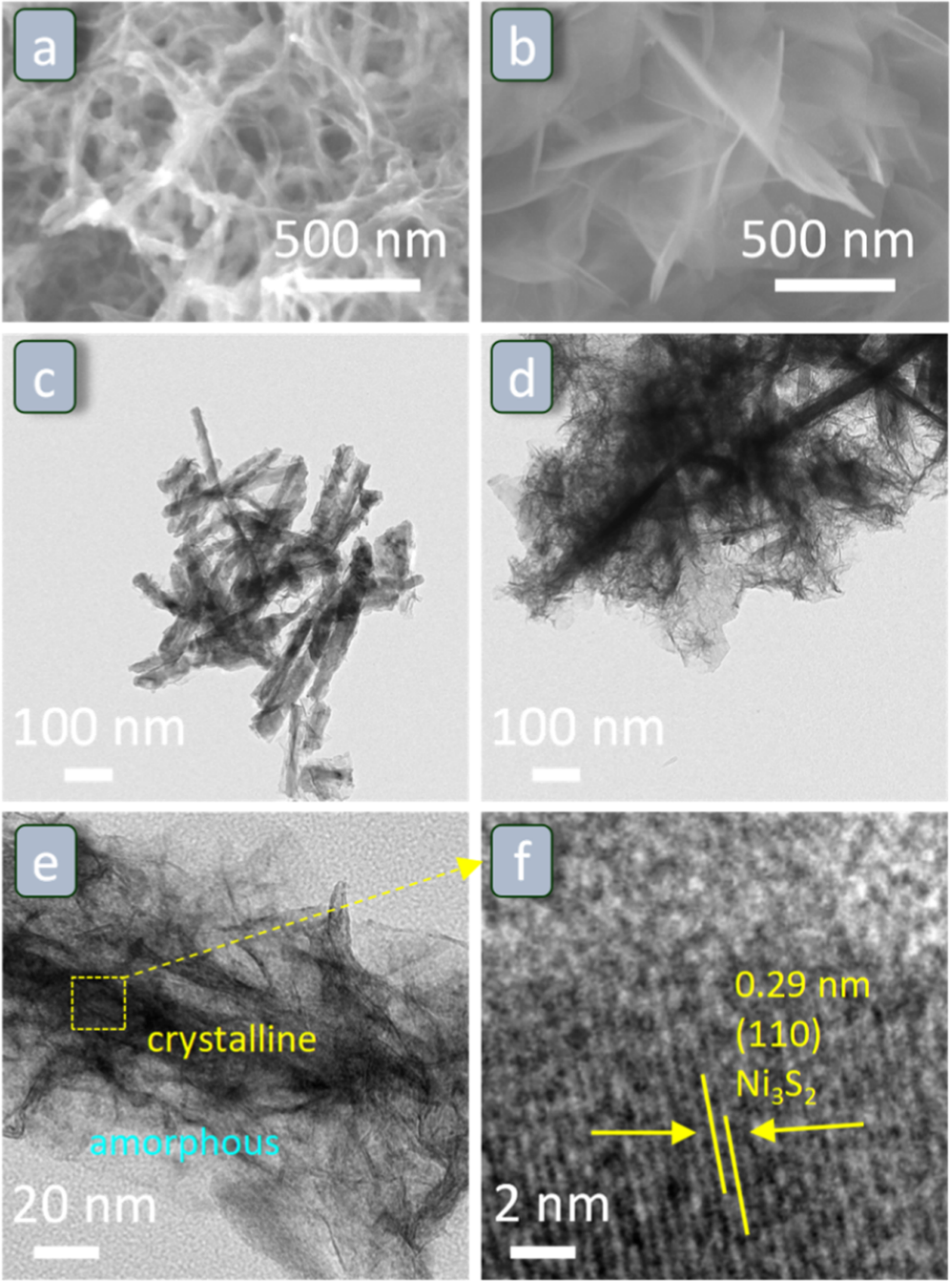
SEM images of (a) Ni_3_S_2_/Ni foam and (b) Fe–Ni_3_S_2_/Ni foam. TEM of nanostructures removed by sonication from (c) Ni_3_S_2_/Ni foam and (d) Fe–Ni_3_S_2_/Ni foam. (e) HRTEM image of the nanostructures removed by sonication from Fe–Ni_3_S_2_/Ni foam and (f) magnified image of the region within the yellow square in (e).

**Figure 5:**
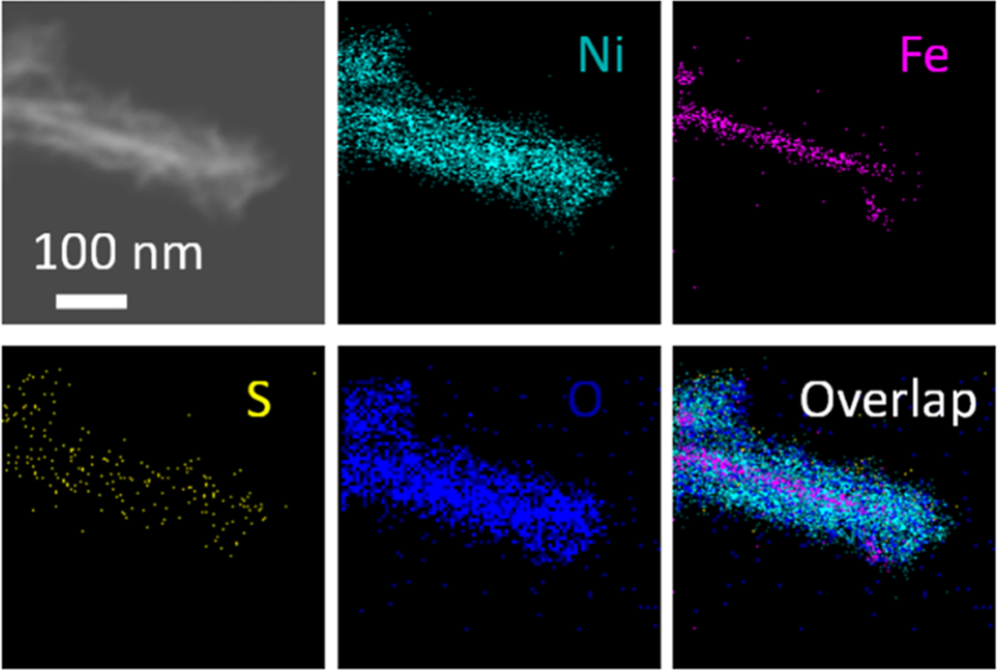
EDX elemental mapping of the nanostructures removed by sonication from Fe–Ni_3_S_2_/Ni foam.

**Figure 6:**
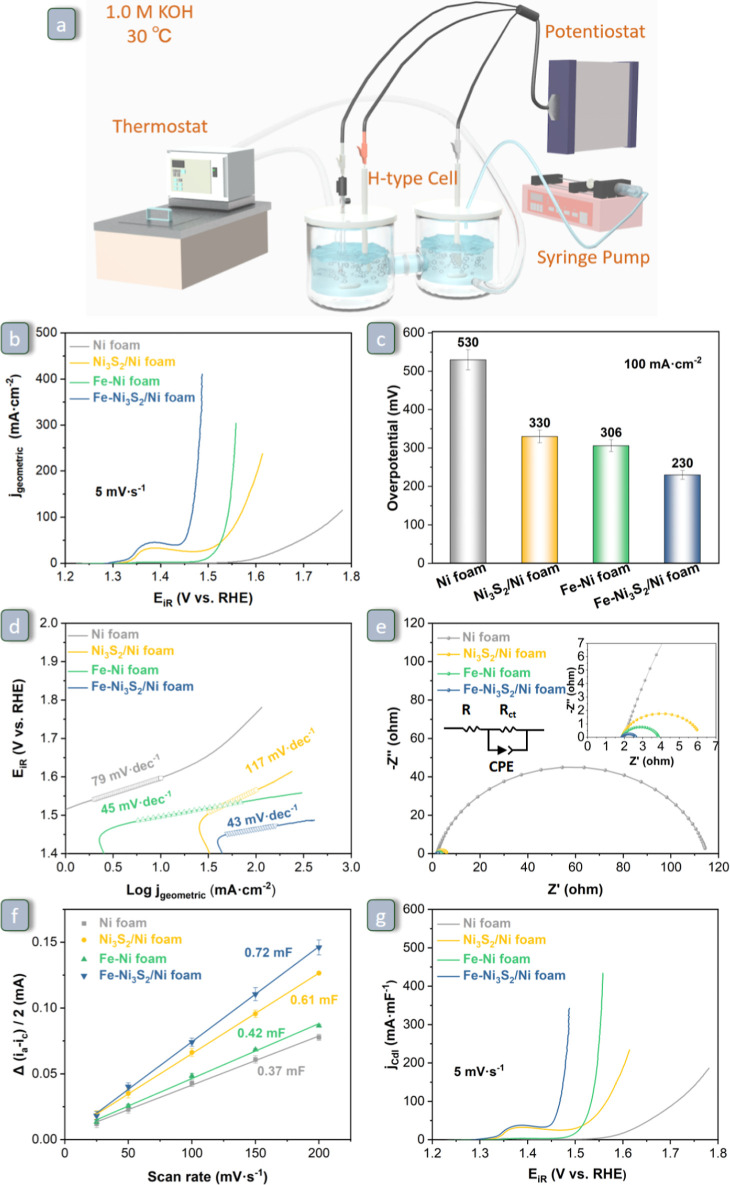
(a) Schematic diagram of the H-type electrolytic cell device with controllable temperature and high current density operation used in this work. (b) *iR*-compensated LSV curves recorded at 5 mV·s^–1^ scan rate, with corresponding (c) OER overpotentials at 100 mA·cm^–2^ and (d) Tafel plots of Ni foam, Ni_3_S_2_/Ni foam, Fe–Ni foam, and Fe–Ni_3_S_2_/Ni foam. (e) Nyquist plots of Ni foam, Ni_3_S_2_/Ni foam, Fe–Ni foam, and Fe–Ni_3_S_2_/Ni foam for OER recorded at an overpotential of 300 mV. The signals at the high-frequency side are enlarged and shown in the inset. (f) *C*_dl_ values (note: these values were normalized by the geometric surface area to facilitate comparison with literature data) and (g) *C*_dl_-normalized LSV curves of Ni foam, Ni_3_S_2_/Ni foam, Fe–Ni foam, and Fe–Ni_3_S_2_/Ni foam. All data were collected in 1.0 M KOH solution as the electrolyte at 30 °C.

**Figure 7:**
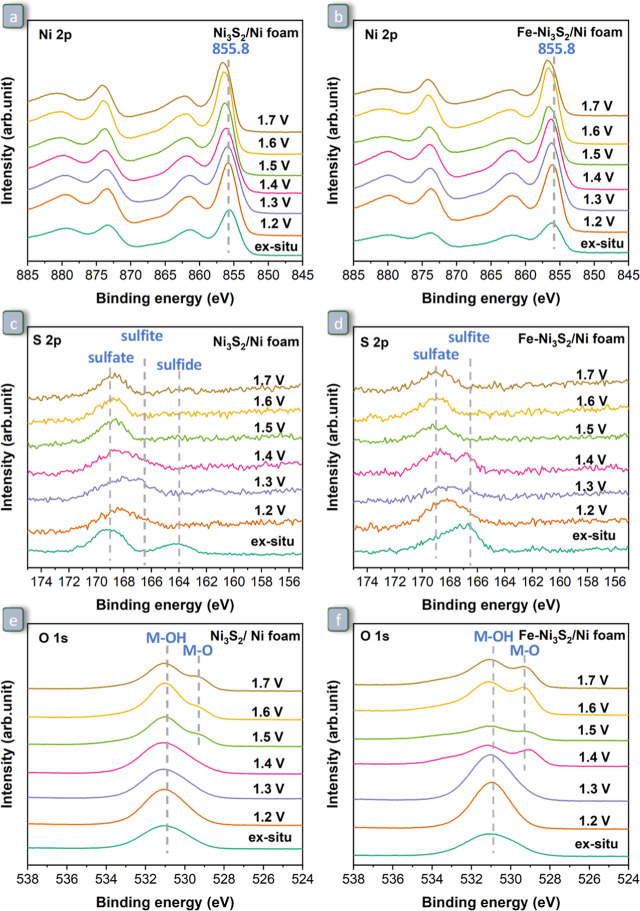
Quasi-in situ XPS signals of Ni_3_S_2_/Ni foam (left) and Fe–Ni_3_S_2_/Ni foam (right): (a,b) Ni 2p; (c,d) S 2p; and (e,f) O 1s core level regions. Electrochemical measurements were conducted in 1.0 M KOH solution at room temperature. All the potentials are indicated in the RHE scale in the figure. The spectra indicated as “ex situ” were collected with as-prepared electrodes before contact with the electrolyte.

**Figure 8:**
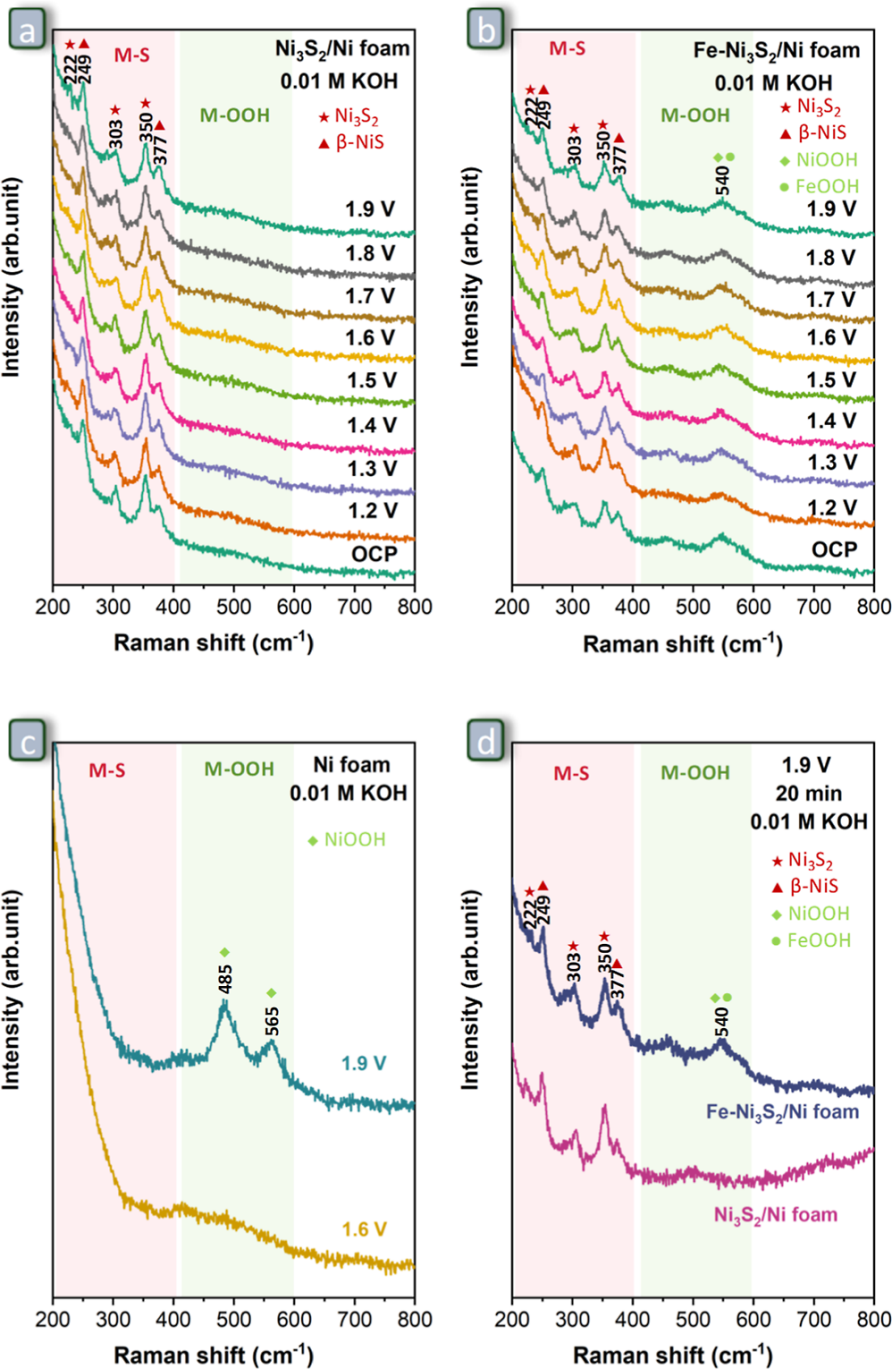
In situ Raman spectra of (a) Ni_3_S_2_/Ni foam, (b) Fe–Ni_3_S_2_/Ni foam, and (c) Ni foam at selected potentials recorded after 30 s. (d) In situ Raman spectra of Ni_3_S_2_/Ni foam and Fe–Ni_3_S_2_/Ni foam recorded after applying potential at 1.9 V vs RHE for 20 min. All measurements were done in 0.01 M KOH solution at room temperature. All the potentials are indicated vs RHE.

**Figure 9:**
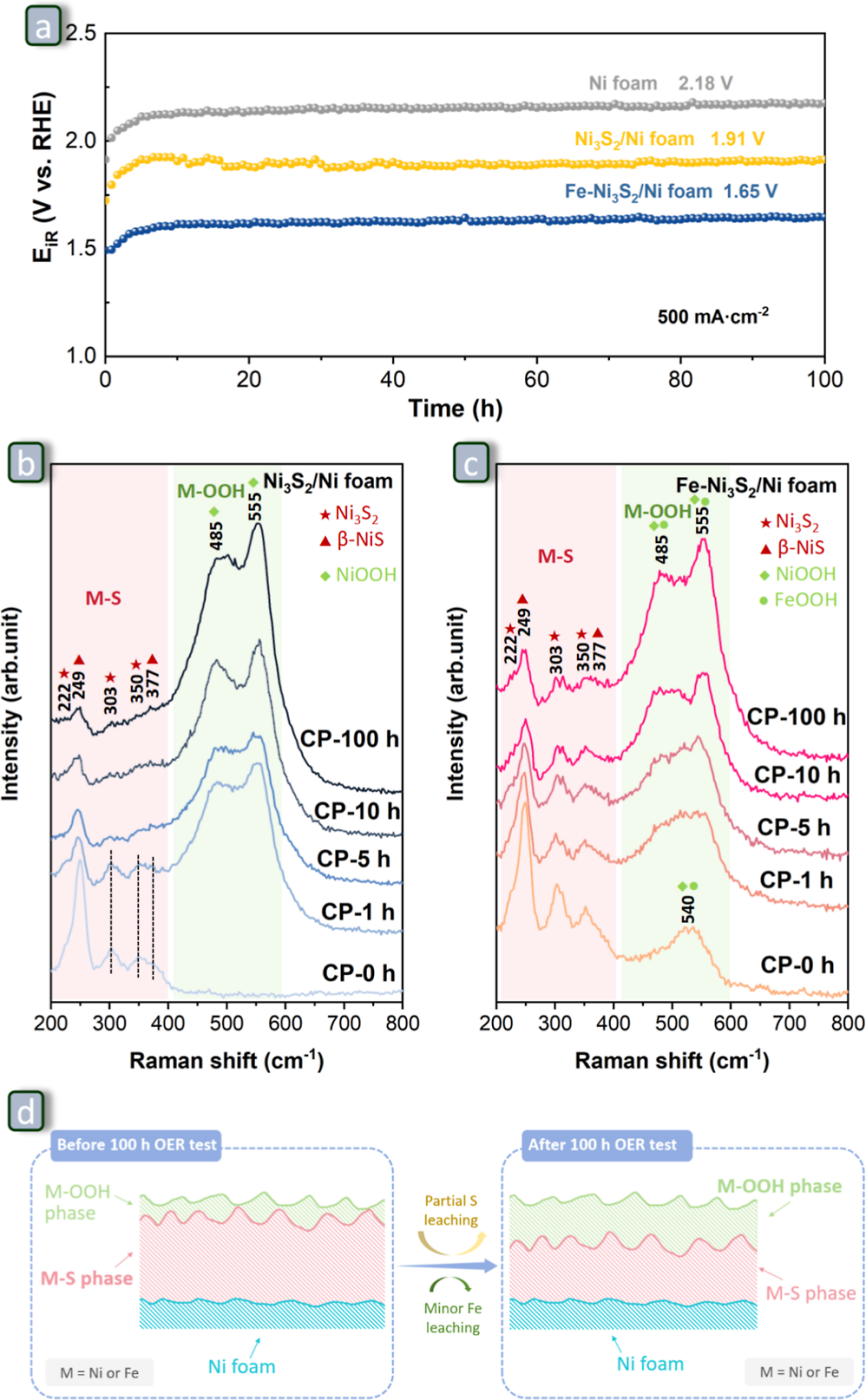
(a) Chronopotentiometric curves of Ni foam, Ni_3_S_2_/Ni foam, and Fe–Ni_3_S_2_/Ni foam at 500 mA·cm^–2^ for 100 h with *iR* compensation in 1.0 M KOH at 30 °C. Raman spectra of (b) Ni_3_S_2_/Ni foam and (c) Fe–Ni_3_S_2_/Ni foam before and after conducting CP for 1, 5, 10, and 100 h. (d) Schematic representation of the proposed evolution of the Fe–Ni_3_S_2_/Ni foam during the 100 h OER test at 500 mA·cm^–2^ in 1.0 M KOH at 30 °C.

**Figure 10:**
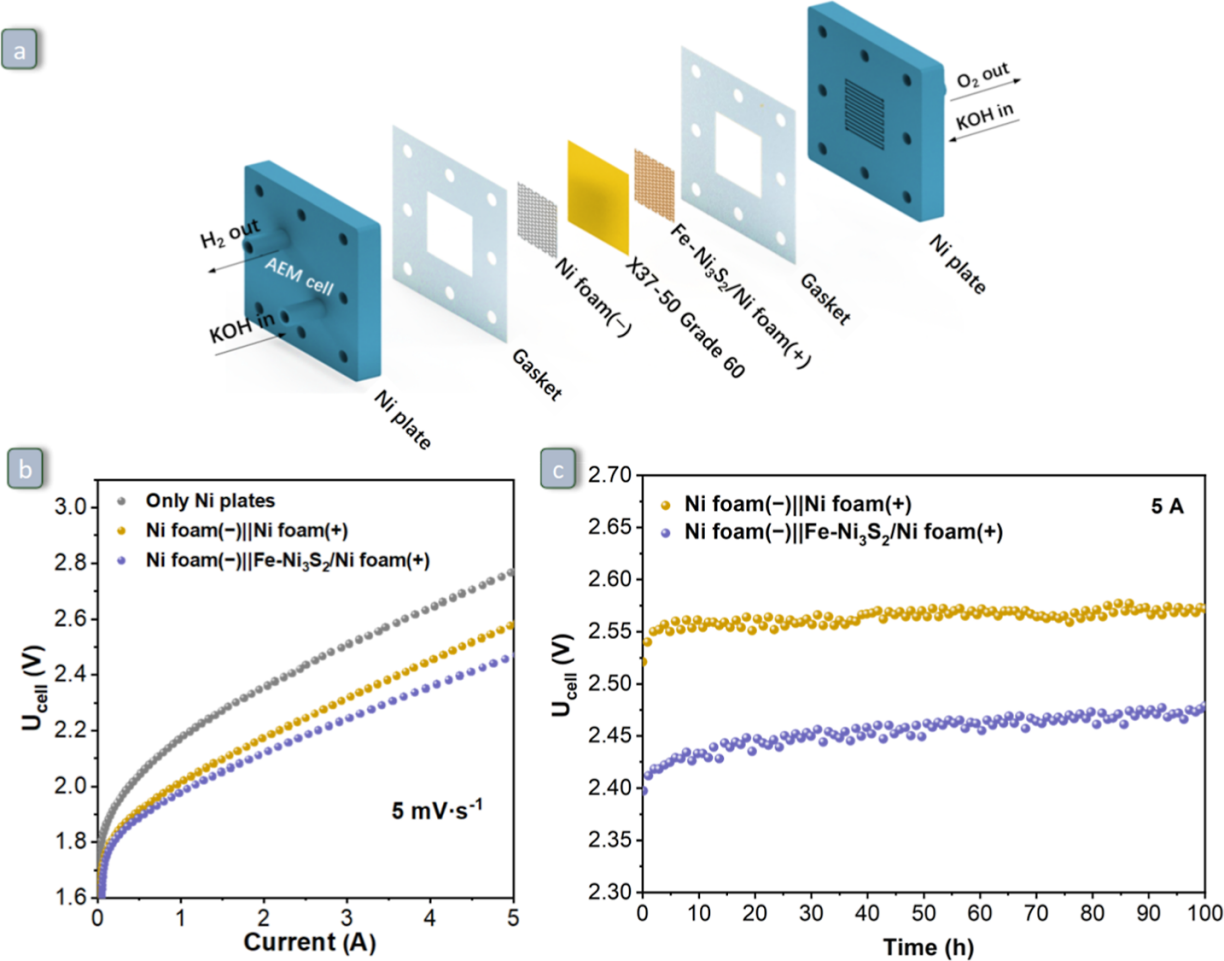
Schematic illustration of the AEM electrolyzer with Ni foam as the cathode and Fe–Ni_3_S_2_/Ni foam as the anode. (b) LSV curves with a scan rate of 5 mV·s^–1^ for two different cell configurations: Ni foam (−) ∥ Ni foam (+) and Ni foam (−) ∥ Fe–Ni_3_S_2_/Ni foam (+), compared to the cell operated with only Ni plates. (c) Chronopotentiometric curves of Ni foam (−) ∥ Fe–Ni_3_S_2_/Ni foam (+) at 5 A for 100 h. All measurements were done in 1.0 M KOH solution at room temperature with a flow rate of 300 mL·min^–1^.

## 3 Results and Discussion

The first step of this work consisted in preparing an OER electrocatalyst by doping Fe into Ni_3_S_2_ nanostructures that were built on Ni foam using a novel synthesis method, with the aim of achieving high electrocatalytic activity and long-term durability when operating at an industrially relevant current density (up to 500 mA·cm^–2^). The method consisted of two steps. First, Ni_3_S_2_ nanothreads with length in the order of hundreds of nanometers and width in the order of tens of nanometers were synthesized on Ni foam (Ni_3_S_2_/Ni foam) by a solvothermal reaction, with S powder as the sulfur source and Ni foam as the nickel source. Then, ferrous sulfate was used as an iron source to obtain nanostructured Fe-doped Ni_3_S_2_ on Ni foam (Fe–Ni_3_S_2_/Ni foam) via a hydrothermal reaction (Figure 1). To evaluate the role of each of the synthetic steps involved in the preparation of Fe–Ni_3_S_2_/Ni foam, two reference electrocatalysts were used in this work: (1) a material prepared by following the procedure described above but without the Fe-doping step (Ni_3_S_2_/Ni foam) and (2) a material in which Fe-containing nanostructures were grown on Ni foam without Ni_3_S_2_ using ferrous sulfate as the iron source (Fe–Ni foam). The prepared materials were characterized by means of a combination of techniques (Section 3.1), and their electrocatalytic performance was thoroughly evaluated by combining voltammetric and chronopotentiometric tests with in situ and post-test characterization (Section 3.2). This approach allowed the definition of property–performance relationships for the novel Fe–Ni_3_S_2_/Ni foam electrocatalyst and rationalizing its enhanced performance compared to the reference electrocatalysts (Ni_3_S_2_/Ni foam, Fe–Ni foam, and Ni foam).

### 3.1 Characterization of the Electrocatalysts

The crystal structure of the as-synthesized materials was investigated by X-ray diffraction (XRD) (Figure 2a). Three main diffraction peaks are observed in the XRD patterns of all materials, located at 44.6, 51.9, and 76.4° and corresponding to the (111), (200), and (220) crystal planes of metallic nickel (JCPDS no. 04-0850), respectively. No additional peaks are present in the diffractogram of Ni foam, whereas for Ni_3_S_2_/Ni foam, the diffraction peaks of hexagonal Ni_3_S_2_ (JCPDS no. 44-1418) are observed at 21.8, 31.4, 38.0, 49.9, 50.4, 55.3, and 55.4°, corresponding to the (101), (110), (003), (113), (211), (122), and (300) planes, respectively. The same peaks due to hexagonal Ni_3_S_2_ were observed in the diffractogram of Fe–Ni_3_S_2_/Ni foam, though weaker in intensity, which indicates that the Fe-doping synthesis step caused a decrease in the crystallinity of the material. The XRD pattern of Fe–Ni foam showed only the signals of metallic Ni from the Ni foam but no diffraction signals of any Fe-based phases, indicating that these are amorphous and/or in too low amount to be detected. Although XRD did not highlight the presence of any Fe-containing crystalline phase, both Fe–Ni_3_S_2_/Ni foam and Fe–Ni foam do contain iron, as demonstrated by different elemental analysis methods (ICP-AES for the overall content and XPS for the surface content, see Figure 3a). Further information about the nickel and iron phases present in the materials can be obtained by Raman spectroscopy (Figure 2b; see also Figure S3 and Table S1 for the position of characteristic peaks for relevant Ni- and Fe-based compounds). The Raman spectrum of the Ni foam does not show any peaks. On the other hand, the spectrum of Fe–Ni foam displays three characteristic peaks positioned at 303, 390, and 680 cm^–1^, which may correspond to α-FeOOH or β-FeOOH.^50^ For Ni_3_S_2_/Ni foam, the observed peaks at 303 and 350 cm^–1^ are the characteristic Ni–S vibrational modes of Ni_3_S_2_,^51,52^ while the peaks at 249 and 377 cm^–1^ correspond to the Ni–S vibrational modes of β-NiS,^53−56^ though this phase was not observed by XRD and thus could be present only in small amount and/or have low crystallinity. The peaks at 249 and 377 cm^–1^ could, in principle, also stem from nickel sulfate (which was observed by XPS, see below). However, the very intense characteristic peak of NiSO_4_ at 980 cm^–1^ was absent in our sample (see Figure S3), making the assignment of the peaks at 249 and 377 cm^–1^ to β-NiS more plausible. The Raman spectrum of Fe–Ni_3_S_2_/Ni foam shows the same peaks in the characteristic zone of the Ni–S vibrational modes as those observed for the parent Ni_3_S_2_/Ni foam. Additionally, a new, very broad, and rather intense band with a maximum at 540 cm^–1^ was observed (Figure 2b, top spectrum). The position of this band and its broad nature suggest that it originates from the overlapping signals of the bending vibration [δ_(Ni–O)_] and stretching vibration [ν_(Ni–O)_] modes in both β- and γ-NiOOH^57,58^ and of the characteristic vibrational modes of α-FeOOH and β-FeOOH,^50^ implying that the Fe-doping process is conducive to the modification of the surface involving formation of NiOOH/FeOOH. This signal and its evolution during the electrochemical tests will be further discussed in Section 3.3.

XPS was used to investigate the surface composition and chemical states of the Ni_3_S_2_/Ni foam and Fe–Ni_3_S_2_/Ni foam (Figure 3). The Ni 2p signal of Ni_3_S_2_/Ni foam (Figure 3b) was deconvoluted into three contributions peaked at binding energies of 852.9 (Ni^0^), 855.8 (oxidized Ni), and 861.1 eV (satellite), with the presence of both Ni^0^ and oxidized Ni species being characteristic of Ni_3_S_2_.^59,60^ The high ratio between the intensity of the peak of oxidized Ni and that of Ni^0^ compared to what is expected based on the stoichiometry of Ni_3_S_2_ (2:1) suggests that the material has undergone partial oxidation, most likely with the formation of surface sulfate species as indicated by the presence of a peak stemming from S–O bonds (besides that due to Ni–S bonds) in the S 2p signal (Figure 3e).^61^ The Ni 2p core level signal of Fe–Ni_3_S_2_/Ni foam shows substantial differences compared to that of Ni_3_S_2_/Ni foam. The binding energy of the oxidized Ni species is shifted to a higher binding energy by 0.3 eV, which indicates that the oxidation state of Ni increased upon the Fe-doping process. In addition, the peak attributed to Ni^0^ that was visible in the Ni 2p signal of Ni_3_S_2_/Ni foam cannot be detected for Fe–Ni_3_S_2_/Ni foam, which suggests that during the synthesis of Fe–Ni_3_S_2_/Ni foam, the Ni_3_S_2_ phase present at the surface of Ni_3_S_2_/Ni foam was converted into more oxidized Ni species. This result is consistent with a decreased overall content of crystalline Ni_3_S_2_ that was earlier observed in Fe–Ni_3_S_2_/Ni foam by XRD and Raman analyses. Fe 2p, the most intense XPS peak of Fe, is unsuitable for quantitative analysis of Fe in the material due to its overlap with the Ni LMM Auger signal (Figure 3c). Yet, the presence and quantity of Fe in Fe–Ni_3_S_2_/Ni foam could be determined from the Fe 3p signal (Figure 3d). The S 2p signal of Ni_3_S_2_/Ni foam shows that this material contains nickel sulfide (Ni–S) bonds and sulfite/sulfate (S–O) bonds,^61^ but after the Fe-doping process, through which Ni_3_S_2_/Ni foam is converted into Fe–Ni_3_S_2_/Ni foam, only S–O bonds remained, and these shifted to slightly higher binding energies, from 167.5 to 168.4 eV (Figure 3e). This implies that the surface sulfides are oxidized as a result of the Fe-doping treatment and also proves the loss in Ni_3_S_2_ crystallinity. Furthermore, the integration of the O 1s signal (Figure 3f and Table S2) of Ni_3_S_2_/Ni foam and Fe–Ni_3_S_2_/Ni foam indicates that the O content increases from 48 to 60 at % as a consequence of the Fe-doping treatment, in agreement with the observed increased oxidation state of Ni. The O 1s signals of the two materials can be deconvoluted in three characteristic peaks: metal–oxygen (M–O) bond at 529.7 eV, metal-hydroxide (M–OH) bond at 531.2 eV, and adsorbed H_2_O at 532.2 eV.^10^ After the introduction of Fe, the ratio between the M–O and M–OH signals increases significantly and reaches a value of M–O/M–OH = 2.2. This suggests the presence of both oxide and oxyhydroxide species in Fe–Ni_3_S_2_/Ni foam, with the formation of the latter being in line with the signal at 540 cm^–1^ in the Raman spectrum of this material (vide supra) and supported by literature reports.^62^ Moreover, the relative ratio of the signal of adsorbed H_2_O slightly increases from 0.25 to 0.32, indicating that the material becomes more hydrophilic. To explain the formation of oxides/oxyhydroxides, we hypothesize that a fraction of the Fe^II^ introduced as FeSO_4_ in the aqueous reaction mixture gets oxidized to Fe^III^ upon reacting with oxygen and water. In turn, the Fe^III^ ions can oxidize Ni^0^ in Ni_3_S_2_ to Ni^II^/Ni^III^, though the extent of the oxidation to Ni^III^ is expected to be thermodynamically limited, as *E*^0^(Fe^III^/Fe^II^) < *E*^0^(Ni^III^/Ni^II^). The formed Fe^III^ and Ni^III^ species can lead to the formation of oxide and oxyhydroxide species. The XPS of Fe–Ni foam (Figure S4) indicates that the Ni surface species of this material have a similar oxidation state as in Fe–Ni_3_S_2_/Ni foam, which is attributed to the interaction with the Fe species. The presence of the latter element was confirmed by the Fe 3p signal (Figure S4). XPS was also used to quantify the atomic ratios of Fe/Ni and S/Ni on the surface of the catalyst. The comparison of the surface analysis by XPS with the overall elemental analysis by ICP-AES shows that in Fe–Ni_3_S_2_/Ni foam Fe and S are mainly present in the surface region of the material (Figure 3a). The same feature is observed for Ni_3_S_2_/Ni foam and Fe–Ni foam (Figure S5).

The synthesis of Ni_3_S_2_ on Ni foam is generally expected to lead to the formation of nanostructures that increase the surface area of the material, which, in turn, is anticipated to lead to enhanced electrocatalytic activity. Therefore, it is important to examine the surface morphology and structure of the as-synthesized materials at different scales by means of scanning electron microscopy (SEM, Figure 4a,b) and transmission electron microscopy (TEM, Figure 4c–f). The SEM images in Figure 4a show that Ni_3_S_2_/Ni foam consists of many interconnected nanothreads forming a web-like structure. The width of the nanothreads was estimated by TEM (Figure 4c) to be 20–40 nm. The length of these nanothreads was between 150 and 400 nm (Figure 4c), but it should be taken into account that this might be an underestimate of the actual length of the nanothreads as the analysis by TEM was carried out on fragments removed from Ni_3_S_2_/Ni foam by sonication (see Experimental Section for more details on the procedure). SEM analysis of the Fe–Ni_3_S_2_/Ni foam (Figure 4b) shows that this material consists of nanosheets instead of the nanothreads observed for the parent Ni_3_S_2_/Ni foam, which indicates that Fe-doping treatment led to morphological changes. The higher magnification images of Fe–Ni_3_S_2_/Ni foam obtained by TEM (Figure 4d) offer a clearer visualization of the nanostructure of this material, in which the nanothreads present in the parent material were covered by thin nanosheets. This more elaborated nanostructure is expected to increase the electrochemical surface area of the material and, thus, its electrocatalytic activity (vide infra). High-resolution TEM (HRTEM) images of Fe–Ni_3_S_2_/Ni foam reveal that the nanosheets are amorphous (Figure 4e), while the nanothreads exhibit the (110) crystal planes of Ni_3_S_2_ with lattice spacings of 0.29 nm (Figure 4f). The same lattice spacings are also observed in the HRTEM image of Ni_3_S_2_/Ni foam (Figure S6). Energy-dispersive X-ray (EDX) spectroscopy elemental analysis of fragments that were ultrasonically removed from the Fe–Ni_3_S_2_/Ni foam (Figures S7 and S8) shows that Ni, Fe, S, and O are not uniformly distributed throughout the material, with some areas being particularly rich in either Fe or S. This non-uniform distribution is attributed to the composite nature of the material consisting of the Ni foam skeleton on which the nickel sulfide nanostructures were grown and eventually modified by the Fe-doping treatment. Some of the fragments (Figure 5) allow us to distinguish between the composition of the nanothreads, which contain Ni, Fe, S, and O, and that of the nanosheets, which consist of Ni, S, and O, but are practically devoid of Fe. It is worth noting that while the treatments that led to the synthesis of Fe–Ni_3_S_2_/Ni foam had a major impact on the surface of the foam at the nanoscale, its porosity at the micrometer scale was not affected (compare the SEM images of the parent Ni foam and of the Fe–Ni_3_S_2_/Ni foam shown in Figure S9).

The morphology of Fe–Ni foam was also investigated, showing markedly different features compared with Fe–Ni_3_S_2_/Ni foam. The surface of Fe–Ni foam presents irregular aggregates of nanoparticles, with the primary nanoparticles being mainly in the 50–150 nm size range (see SEM and TEM images in Figure S10).

### 3.2 Electrocatalytic OER Performance

As evidenced in the previous section, our novel Fe–Ni_3_S_2_/Ni foam material displays several characteristics that make it promising for application as an electrocatalyst for the OER: (i) the presence of Fe and Ni, which are known to be active in promoting the OER, and (ii) a nanostructured surface, which is expected to be beneficial to the electrocatalytic performance by providing a large electrochemically active surface area. In order to investigate if these promising physicochemical features led to the desired electrocatalytic OER performance, we performed a thorough study in an H-type electrolytic cell (Figure 6a) using a combination of electrochemical techniques: linear sweep voltammetry (LSV), cyclic voltammetry (CV), electrochemical impedance spectroscopy (EIS), and chronopotentiometry (CP). All these tests were carried out in 1.0 M KOH at 30 °C. Following a common trend in the literature, the LSV curves were corrected based on the values of the uncompensated resistance (Figure 6b) determined by EIS. Though plotting the logarithm of these curves (Figure S11a) indicates that no obvious overcompensation occurred, for completeness, the LSV curves without *iR* compensation and the uncompensated resistances of the as-synthesized electrocatalysts are provided in the Supporting Information (Figure S11b,c). The *iR*-corrected LSV curves in Figure 6b show that Fe–Ni_3_S_2_/Ni foam outperforms Ni_3_S_2_/Ni foam, Fe–Ni foam, and Ni foam in terms of OER overpotential and current density. More specifically, the overpotential at a current density of 100 mA·cm^–2^ (Figure 6c) and the Tafel slope (Figure 6d) of Fe–Ni_3_S_2_/Ni foam (230 mV, 43 mV·dec^–1^) are markedly lower than those of Ni_3_S_2_/Ni foam (330 mV, 117 mV·dec^–1^), Fe–Ni foam (306 mV, mV·dec^–1^), and Ni foam (530 mV, 79 mV·dec^–1^). The lower overpotential of Fe–Ni_3_S_2_/Ni foam means that this electrocatalyst requires a lower electric power input to sustain the reaction at the chosen current density, and the lower Tafel slope of Fe–Ni_3_S_2_/Ni foam implies faster reaction kinetics. Though comparison with electrocatalysts in the literature should be done with caution, as the OER performance does not depend only on the intrinsic activity of the electrocatalyst but also on the features of the electrochemical cells and the operating conditions, Table S3 shows that Fe–Ni_3_S_2_/Ni foam is among the most active NiFe-based OER electrocatalysts in terms of low overpotential and Tafel slope. Furthermore, the mass activity and turnover frequency (TOF) were calculated (Figure S12). Notably, Fe–Ni_3_S_2_/Ni foam still exhibits the best electrocatalytic performance in terms of overpotential needed to reach a current density of 10 mA·mg^–1^ (Figure S12a) and a TOF of 0.5 s^–1^ (Figure S12b).

To further examine the OER kinetics, EIS was carried out at an overpotential of 300 mV. From the Nyquist plots drawn by constructing an equivalent circuit (Figure 6e), the charge-transfer resistance (*R*_ct_) was determined for Fe–Ni_3_S_2_/Ni foam (0.77 Ω) and found to be much smaller than those of Ni_3_S_2_/Ni foam (4.26 Ω), Fe–Ni foam (1.98 Ω), and Ni foam (51.84 Ω). These data indicate that the Fe–Ni_3_S_2_/Ni foam displays a higher electron-transfer rate, leading to the faster reaction kinetics demonstrated by the Tafel slope. It is worth noting that the remarkably high charge-transfer resistance value observed for Ni foam compared to the other electrocatalysts (Figure 6e) is due to the fact that on this material, the OER is virtually not taking place at an overpotential of 300 mV (i.e., 1.53 V vs RHE, see Figures 5b and S8a) at which the EIS analysis was carried out. To characterize the Ni foam also at a potential at which OER occurs, the EIS analysis of Ni foam and Fe–Ni_3_S_2_/Ni foam was also performed at an overpotential of 400 mV. The results (Figure S11d) show that also under these conditions, the *R*_ct_ of Fe–Ni_3_S_2_/Ni foam (0.29 Ω) is significantly lower than that of Ni foam (3.37 Ω).

The electrochemically active surface area (ECSA) is an important factor that influences the apparent activity of the electrocatalyst. The ECSA is typically estimated by measuring the double-layer capacitance (*C*_dl_), although for this purpose, the specific capacitance of the material (*C*_s_) would need to be known (ECSA = *C*_dl_/*C*_s_). The double-layer capacitances (*C*_dl_) of the as-synthesized electrocatalysts were determined by CV measurements in the non-Faradaic potential range (Figure S13). As shown in Figure 6f, the *C*_dl_ value of the Fe–Ni_3_S_2_/Ni foam (0.72 mF) is higher than those of the Ni_3_S_2_/Ni foam (0.61 mF), Fe–Ni foam (0.42 mF), and Ni foam (0.37 mF). Although caution is advised in the interpretation of this trend because the specific capacitance may differ among these materials, the fact that the nanostructured Fe–Ni_3_S_2_/Ni foam and Ni_3_S_2_/Ni foam materials display significantly higher *C*_dl_ values compared to their counterparts without sulfides is likely to indicate that these electrocatalysts have a larger electrochemically active surface area in contact with the electrolyte, thereby contributing to their observed higher OER activity (Figure 6b). This result highlights the positive effect of the nanostructuring of the electrocatalysts (Figure 4) on their OER performance. However, this is not the only feature of our materials that affects their electrocatalytic activity, which also depends on their chemical composition. To better evaluate this aspect, the currents measured during the LSV tests were normalized with respect to the *C*_dl_ values (Figure 6g). By comparing Figure 6b (current normalized by geometric area) and Figure 6g (current normalized by double-layer capacitance), Fe–Ni_3_S_2_/Ni foam still exhibits the best electrocatalytic performance (in terms of overpotential needed to reach a current density of 100 mA·mF^–1^), suggesting that the enhanced activity of Fe–Ni_3_S_2_/Ni foam compared to the other electrocatalysts is not only due to its higher ECSA but likely also due to a higher intrinsic activity of the sites at the surface of this material. Based on the characterization data discussed above, it can be inferred that these more active sites are related to the introduction of Fe into Ni_3_S_2_/Ni foam, in line with the beneficial effect that Fe species have been reported to exert on Ni-based electrodes for alkaline OER.^24,29,47,63^ Although the promoting effect of iron species has been established, it is worth noting that the exact role of these species and the mechanism through which this occurs have not yet been fully elucidated.^64^ In our work, the beneficial effect of Fe is further demonstrated by the higher *C*_dl_-normalized current densities achieved by Fe–Ni foam compared to Ni_3_S_2_/Ni foam and Ni foam (Figure 6g).

### 3.3 Effect of the Applied Potential on the Surface Structure of the Electrocatalysts

The two most promising electrocatalysts identified in this study, Fe–Ni_3_S_2_/Ni foam and Ni_3_S_2_/Ni foam, are characterized by different phases at their surfaces (see Section 3.1). To understand their electrocatalytic behavior, it is useful to monitor the stability of these phases as a function of the applied potential. For this purpose, the two materials were investigated by quasi-in situ XPS (Figure 7) and in situ Raman spectroscopy (Figure 8). First, the effect of the applied potential on the surface species throughout the OER process was monitored by quasi-in situ XPS (Figure 7). As the electrode potential increases (Figure 7a,b), the Ni 2p core level signal of Ni_3_S_2_/Ni foam and Fe–Ni_3_S_2_/Ni foam gradually shifts to a higher binding energy, from 855.6 to 856.4 eV for the former and from 856.1 to 856.6 eV for the latter. These shifts are in agreement with the oxidation of Ni^II^ to Ni^III^ observed by LSV between 1.35 and 1.4 V vs RHE (Figure 6b). Notably, the Ni 2p signal of Fe–Ni_3_S_2_/Ni foam at all potentials below the oxidation of Ni^II^ to Ni^III^ (i.e., *E* ≤ 1.3 V vs RHE) peaks at a higher binding energy (856.2 eV) than that of Ni_3_S_2_/Ni foam (855.9 eV), see Figure 7a vs Figure 6b. This suggests that the presence of Fe^III^ in the proximity of Ni^II^ decreases the electron density of the latter. The Fe 2p signal in Fe–Ni_3_S_2_/Ni foam indicates that iron mainly exists as Fe^III^ without obvious changes over the entire potential range (Figure S14).^65^ For both Ni_3_S_2_/Ni foam and Fe–Ni_3_S_2_/Ni foam, the S 2p signal has a low signal-to-noise ratio (Figure 7c,d), corresponding to the rather small S content on the surface, as already observed in the parent samples (Figure 3f). Before the electrochemical testing, sulfides and sulfites/sulfates are present on Ni_3_S_2_/Ni foam, and only sulfites/sulfates are present on Fe–Ni_3_S_2_/Ni foam (vide supra).^66,67^ The evolution of the S 2p core level signal upon increasing the applied potential indicates that the sulfide species on Ni_3_S_2_/Ni foam tend to oxidize to sulfites and eventually to sulfates (Figure 7c), while sulfites on Fe–Ni_3_S_2_/Ni foam are oxidized to sulfates (Figure 7d). Also the O 1s core level signal changes with the applied potential: at ≥1.5 V, the relative intensity of the contribution due to metal oxide (M–O) bonds in Ni_3_S_2_/Ni foam (Figure 7e) increases substantially compared to that assigned to metal hydroxide (M–OH) bonds, while in Fe–Ni_3_S_2_/Ni foam (Figure 7f), the relative intensity of the M–O band is more prominent and its presence is already observable at 1.4 V vs RHE.^68^ Based on Raman spectroscopy analysis (vide infra) and according to previous literature reports, the increase in the relative intensity of the M–O contribution is attributed to the formation of oxyhydroxide phases (M-OOH, with M = Ni or Fe).^62^

In situ Raman spectroscopy was utilized to monitor the changes in phases present at the surface of the Ni_3_S_2_/Ni foam and Fe–Ni_3_S_2_/Ni foam during the OER at different potentials (Figure 8). At the open-circuit potential (OCP), the Raman spectrum of Ni_3_S_2_/Ni foam (Figure 8a) displayed four peaks in the 200–400 cm^–1^ region assigned to metal sulfides (M–S),^51,56,69^ whereas the spectrum of Fe–Ni_3_S_2_/Ni foam (Figure 8b) additionally shows a peak at 550 cm^–1^ attributed to metal oxyhydroxides (M–OOH).^50,58,70^ This is fully in agreement with the ex situ Raman analysis presented in Figure 2b (vide supra). More details can be found in Figure S3 and Table S1 for the position of characteristic peaks for relevant NiFe-based compounds. As the applied potential is increased stepwise from 1.2 to 1.9 V vs RHE, the in situ Raman spectra of Ni_3_S_2_/Ni foam (Figure 8a) and Fe–Ni_3_S_2_/Ni foam (Figure 8b) do not change substantially. On the other hand, the in situ Raman spectrum of Ni foam (Figure 8c) does not show any peak at 1.2 V vs RHE, but two peaks at 485 and 565 cm^–1^ corresponding to NiOOH are found at 1.9 V.^70,71^ Moreover, Ni_3_S_2_/Ni foam and Fe–Ni_3_S_2_/Ni foam do not show an obvious phase change, even when being kept at 1.9 V vs RHE for a longer time (20 min, Figures 8d and S15a,b). This suggests that under the relatively mild conditions in which these tests were carried out (0.01 M KOH), Ni sulfides in Ni_3_S_2_/Ni foam and Fe–Ni_3_S_2_/Ni foam are more stable than the Ni species present in the near-surface region of Ni foam, which are expected to include a substantial fraction of metallic Ni and thus be more prone to undergo oxidative changes. After reaching 1.9 V vs RHE of applied potential, the electrode was brought back to 1.2 V vs RHE, and the in situ Raman spectra of Ni_3_S_2_/Ni foam (Figure S15c) and Fe–Ni_3_S_2_/Ni foam (Figure S15d) were collected again. These spectra do not show any obvious change compared to the spectra recorded after increasing the potential from OCP to 1.2 V vs RHE (Figure S15c,d), further demonstrating the stability of Ni_3_S_2_/Ni foam and Fe–Ni_3_S_2_/Ni foam under the tested conditions. Furthermore, the Raman spectra of Ni_3_S_2_/Ni foam and Fe–Ni_3_S_2_/Ni foam after a chronopotentiometric test under harsher conditions (1.0 M KOH, 500 mA·cm^–2^, 100 h) were measured to monitor their phase transformation (see Section 3.4).

### 3.4 Long-Term Stability Tests

For a promising OER electrocatalyst, it is crucial to have excellent long-term stability at an industrially relevant high current density (for example, 500 mA·cm^–2^).^2,4,5^ One of the challenges when measuring the stability of an OER catalyst at high current densities using a laboratory-scale H-type electrolytic cell to assess the activity is related to the fact that a large amount of water will be converted into hydrogen and oxygen. If the consumed water is not replenished, the liquid level will gradually decrease, which on the one hand may lead to substantial changes in the pH and on the other hand will eventually cause the electrode to be disconnected from the electrolyte. To overcome this issue, a syringe pump was used to replenish the consumed water at a constant rate of 3.0 ± 0.5 μL·min^–1^, which was tuned to the rate of the reaction (see Figure 6a and Experimental Section). This allowed testing the durability of Ni foam, Ni_3_S_2_/Ni foam, and Fe–Ni_3_S_2_/Ni foam at 500 mA·cm^–2^ for prolonged time (100 h). All three electrocatalysts were able to drive the OER at this industrially relevant current density, displaying an initial minor deactivation, followed by a constant performance for the rest of the test (Figures 9a and S16a). Although the long-term stability profile is similar for the three electrocatalysts, it is important to underline that the overpotential of the Fe–Ni_3_S_2_/Ni foam is always substantially lower than that of Ni_3_S_2_/Ni foam and Ni foam, in agreement with the trend observed by LSV (Figure 6b). Despite the excellent long-term stability displayed by these electrocatalysts, it is worth noting that a small amount of a dark powder was discovered at the bottom of the electrolytic cell after the durability test for the Ni_3_S_2_/Ni foam and Fe–Ni_3_S_2_/Ni foam. The XRD pattern (Figure S16b) of the black powder collected after the durability test of Fe–Ni_3_S_2_/Ni foam exhibits only one broad peak, while the corresponding Raman spectrum (Figure S16c) shows no signal, and the amount was too small for determining the composition, making it difficult to identify the species contained in this powder residue.

To explain the difference in electrocatalytic performance under operating conditions between Ni_3_S_2_/Ni foam and Fe–Ni_3_S_2_/Ni foam, their Raman spectra were collected before and after the OER in 1.0 M KOH for 1, 5, 10, and 100 h (labeled CP-0 h, CP-1 h, CP-5 h, CP-10 h, and CP-100 h, respectively). As the reaction time increased (Figure 9b,c), for both Ni_3_S_2_/Ni foam and Fe–Ni_3_S_2_/Ni foam, the intensity of the peaks located at 200–400 cm^–1^, representing metal sulfide (MS),^51,56,69^ can be seen to gradually weaken, while that of the peaks located at 400–600 cm^–1^, corresponding to metal oxyhydroxides (M–OOH),^50,58,70^ increased. Compared to Fe–Ni_3_S_2_/Ni foam, the peaks observed at 200–400 cm^–1^ of Ni_3_S_2_/Ni foam disappeared at an earlier stage, with only one peak still being observed after 5 h of reaction time (Figures 9b,c, and S17), attributed to MS. This observation may indicate that the presence of Fe helps to hinder the corrosion of sulfide during the OER process. Similar findings were reported by Zhang et al., who demonstrated that Fe bonded to S in the bulk can act as a sacrificial agent, mitigating the oxidative corrosion of some of the Ni–S bonds, as revealed by XPS fitting analysis.^35^ Normally, the bands at 485 and 555 cm^–1^ are attributed to the bending and stretching modes of Ni–O in NiOOH. Differences in the relative intensity between the band at 485 cm^–1^ and that at 555 cm^–1^ has been correlated to the relative abundance of γ-NiOOH and β-NiOOH phases.^70,71^ However, the fact that in our spectra the band at 555 cm^–1^ is more intense than that at 485 cm^–1^ does not correspond to any previous report, suggesting that other species present in our materials might contribute to the observed signal. In materials containing FeOOH, an increasing content of this compound has been reported to cause an increase in the relative intensity of the band at 555 cm^–1^,^71^ and this might account for the observed ratio between the bands at 485 and 555 cm^–1^ in the spectra of Fe–Ni_3_S_2_/Ni foam (Figure 9c). However, the similarity of the Raman spectrum of Ni_3_S_2_/Ni foam and that of Fe–Ni_3_S_2_/Ni foam suggests caution in drawing such conclusion. Based on these considerations, Raman spectroscopy does not allow defining unequivocally which NiOOH/FeOOH phases were present on Fe–Ni_3_S_2_/Ni foam after the 100 h durability test.

To further reveal possible changes in Fe–Ni_3_S_2_/Ni foam after the 100 h durability test, the material was characterized by SEM, TEM, XPS, and ICP-AES. The nanosheets appeared curled up (Figure S18a,b) contrary to the fresh material, and the nanothreads were almost completely converted into nanosheets (Figure S18c), indicating a certain degree of reorganization of the surface. Despite these changes, blurred lattice fringes with 0.29 nm spacing corresponding to the (110) crystal planes of Ni_3_S_2_ were still visible (Figure S18d), proving that the Ni_3_S_2_ phase was preserved, though with lower crystallinity. EDX elemental mapping showed that Ni, Fe, S, and O were not distributed uniformly (Figures S19 and 20), as also observed in the fresh material (Figures S7 and 8), but the average S content was lower in the used catalyst. In the XPS spectra, the Ni 2p (Figure S21a), Fe 2p_3/2_ (Figure S21b), and Fe 3p (Figure S21c) core level regions were shifted toward higher binding energies after the 100 h durability test, pointing to the presence of Ni and Fe in higher oxidation states. The intensity of the S 2p signal (Figure S21d) was considerably lower after the durability test, implying a loss of sulfur during the reaction. Additionally, the O 1s peak (Figure S21e) exhibited a shift to higher binding energies, which might indicate a higher degree of hydration of the surface. The Fe/Ni and S/Ni surface atomic ratios of the Fe–Ni_3_S_2_/Ni foam before and after the durability test were estimated based on the XPS spectra and showed that the relative Fe and S content decreased during the test (Figure S22a). The same trend was observed by ICP-AES (Figure S22b). Furthermore, the ICP-AES data also showed that the S concentration in the electrolyte increased from 0 to 78 ppm, demonstrating that S was released into the electrolyte during the chronopotentiometric test. On the other hand, no Fe was found in the electrolyte, indicating that Fe leaching, if any, was below the detection limit of ICP-AES. In summary, based on the physicochemical characterization, we can conclude that after the 100 h OER durability test, the nanostructure of the Fe–Ni_3_S_2_/Ni foam slightly changed. While Ni_3_S_2_ was still present in the sample, its crystallinity was lower than in the as-prepared samples, and the NiFe-based oxyhydroxide phases became more prominent (Figure 9d). Since Ni_3_S_2_ is expected to ensure good electrical conductivity and the NiFe-based oxyhydroxide can provide active sites for the OER, the material investigated after a 100 h durability test displays desirable features for promoting the OER. Therefore, despite the minor loss of Fe and the more significant one of S, Fe–Ni_3_S_2_/Ni foam maintained a rather stable electrocatalytic performance (Figure 9a). This is consistent with iron being an active species for OER and surface S-containing species being the precursor for Fe and Ni (oxy)hydroxides.

The performance and long-term stability of Fe–Ni_3_S_2_/Ni foam was further investigated and validated by carrying out water splitting in a 5 cm^2^ AEM electrolyzer operating in flow mode (Figure 10a). All measurements were performed in 1.0 M KOH solution at room temperature with a flow rate of 300 mL·min^–1^. For these tests, Ni foam was used as the cathode, while for the anode, either Fe–Ni_3_S_2_/Ni foam or Ni foam was used. It is worth noting that the casing of the AEM electrolyzer consists of Ni plates (Figure 10a), and it is thus expected to contribute to the overall activity in water splitting. Therefore, the LSV curve of the AEM electrolyzer with only the Ni plates but without additional Ni foam-based electrodes was also measured. As shown in Figure 10b, Fe–Ni_3_S_2_/Ni foam outperformed Ni foam and the cell with only the Ni plates in terms of the cell voltage (*U*_cell_) and current. Additionally, the AEM electrolyzer consisting of Ni foam (−) ∥ Fe–Ni_3_S_2_/Ni foam (+) showed nearly stable operation for 100 h at 5 A, with the cell voltage always being lower than that observed with the Ni foam (−) ∥ Ni foam (+) configuration (Figure 10c). Here, the current was reported rather than the current density because the casing of the AEM electrolyzer consisting of Ni also contributes to the electrolysis of water, making it difficult to define the geometric surface area. These findings highlight the promising qualities of Fe–Ni_3_S_2_/Ni foam for potential application in industrial water electrolysis. Future work could aim at extending the stability tests to investigate the effect of higher temperatures (e.g., 60 to 80 °C) and of more concentrated electrolytes (e.g., 7 M KOH).

## 4 Conclusions

In this work, we synthesized an Fe-doped Ni_3_S_2_ electrocatalyst on Ni foam through a two-step procedure involving a solvothermal reaction to generate the nickel sulfide phase, followed by a hydrothermal reaction to introduce Fe species in the material. The obtained Fe–Ni_3_S_2_/Ni foam was evaluated as an electrocatalyst for the OER, which is the anodic reaction in water electrolysis. The combined presence of Ni and Fe species was expected to create highly active sites for OER in alkaline environment, and the nanostructured surface was anticipated to provide a large electrochemically active surface area. Indeed, these features endowed the obtained material, Fe–Ni_3_S_2_/Ni foam, with a high electrocatalytic OER activity in the 1.0 M KOH electrolyte, with a low overpotential of 230 mV at 100 mA·cm^–2^ and a low Tafel slope of 43 mV·dec^–1^. Importantly, Fe–Ni_3_S_2_/Ni foam displayed a stable performance at industrially relevant current density (500 mA·cm^–2^) with an electrode potential of 1.65 V vs RHE over 100 h chronopotentiometry, despite a small amount of S and Fe being lost in this process. The electrocatalytic performance of Fe–Ni_3_S_2_/Ni foam, in terms of activity and stability, was superior to Ni foam and to two reference materials prepared with the same approach but either without introducing Fe species (Ni_3_S_2_/Ni foam) or without introducing the Ni sulfide phase (Fe–Ni foam). The enhanced OER performance of the Fe–Ni_3_S_2_/Ni foam electrocatalyst was elucidated by thorough characterization with a combination of techniques, including (quasi) in situ monitoring by XPS and Raman spectroscopy of the phases present in the electrocatalyst as a function of the applied potential. Based on these characterization studies, it was concluded that the OER activity and stability of the Fe–Ni_3_S_2_/Ni foam electrocatalyst stem from a combination of three features: (a) a nanostructured surface consisting of nanothreads decorated with nanosheets grown directly on the Ni foam, leading to high electrochemically active surface area and displaying good stability under the operating conditions, (b) the presence of surface Ni–Fe oxyhydroxide species, providing actives sites for the OER, and (c) a relatively stable nickel sulfide phase that is expected to endow high electrical conductivity. Finally, a 5 cm^2^ AEM flow electrolyzer was used to validate the activity and stability of the Fe–Ni_3_S_2_/Ni foam as a cathode for water splitting, leading to a promising stable performance at 5 A for 100 h at a cell potential of 2.45 V.

## Supporting Information Available

Experimental data, potential difference between the HgHgOKOH (1 M) electrode and the RHE, XPS signals, Raman spectra, characteristic Raman peak positions, XPS fitting analysis, XPS signals, Fe/Ni and S/Ni atomic ratios, HRTEM images, EDX elemental mapping images, Fe/Ni and S/Ni atomic ratios, SEM images, LSV curves, OER performance, mass activity and TOF, whole mass and mass ratio of the electrodes, CV curves, and CP curves (DOCX)
