# Supplementary material for: Nanostructured Fe-Doped Ni3S2 Electrocatalyst for the Oxygen Evolution Reaction with High Stability at an Industrially-Relevant Current Density
Source: ACS Appl Mater Interfaces. 2024 Oct 15;16(43):58520–35. doi: 10.1021/acsami.4c09821 (PMC11533162; doi:10.1021/acsami.4c09821)
Supplement: Supplementary file 1 — am4c09821_si_001.docx [file am4c09821_si_001.docx]

# Supporting Information

Nanostructured Fe-Doped Ni_3_S_2_ Electrocatalyst for the Oxygen Evolution Reaction with High Stability at Industrially-Relevant Current Density

Jiahui Zhu ^a^, Wei Chen ^b^, Stefano Poli ^a^, Tao Jiang ^a^, Dominic Gerlach ^c^, João R. C. Junqueira ^d^, Marc C. A. Stuart ^e^_,_ Vasileios Kyriakou ^a^, Marta Costa Figueiredo ^b^, Petra Rudolf ^c^, Matteo Miola ^a^, Dulce M. Morales ^a^, Paolo P. Pescarmona ^a,^ *

^a^ Chemical Engineering group, Engineering and Technology institute Groningen (ENTEG), University of Groningen, The Netherlands
^b^ Department of Chemical Engineering and Chemistry, Eindhoven University of Technology, The Netherlands

^c^ Zernike Institute for Advanced Materials, University of Groningen, The Netherlands

^d^ Analytical Chemistry - Center for Electrochemical Sciences (CES), Faculty of Chemistry and Biochemistry, Ruhr University Bochum, Germany

^e^ Electron Microscopy Group, Groningen Biomolecular Sciences and Biotechnology Institute, University of Groningen, The Netherlands

* Corresponding author: p.p.pescarmona@rug.nl

Fig. S1. The difference between the Hg$|$HgO$|$KOH (1 M) electrode and the reversible hydrogen electrode (RHE, Gaskatel) at 30 °C in 1.0 M KOH aqueous solution for 100 s.

Fig. S2. XPS signals of the (a) survey spectra, (b) C 1s, (c) O 1s, (d) Ni 2p and (e) Fe 2p core level regions of Vulcan XC 72 carbon powder.

Fig. S3. Raman spectra of Ni_3_S_2_/Ni foam, Fe-Ni_3_S_2_/Ni foam, NiSO_4_·6H_2_O (ACS reagent, ≥ 98.0%, Sigma-Aldrich), and FeSO_4_·7H_2_O (ACS reagent, ≥ 99.0%, Sigma-Aldrich).

Table S1. Characteristic Raman peak positions of possible nickel- and iron-based compounds present in the materials reported in this work. Bold characters indicate the peaks observed in the spectra of Ni_3_S_2_/Ni foam and Fe-Ni_3_S_2_/Ni foam.

| Compounds | Raman peak positions (cm^-1^) | Reference |
| --- | --- | --- |
| **Ni_3_S_2_** | **188**, 202, **224**, **304**, 325, **350** | ^1^ |
| α-NiS | 147, 166, 175, 222, 285, 333, 375 | ^2^ |
| β-NiS | 149, 208, 230, **247**, 250, 300, 352, **373** | ^3^ |
| NiS_2_ | 274, 285, 479 | ^2^ |
| Ni_3_S_4_ | 223, 287, 338, 380 | ^2^ |
| **NiSO_4_·6H_2_O** | 206, **244**, **376**, 438, 466, 603, 987, 1087, 1123 | ^4^ |
| **NiSO_4_·6H_2_O** | 212, **249**, **377**, 440, 470, 615, 990, 1093, 1138 | This work |
| FeSO_4_·H_2_O | 112, 158, 218,271, 298, 423, 492, 615, 623, 661, 850, 1018, 1073, 1092, 1194, 1478, 3137, 3246, 3410 | ^5^ |
| FeSO_4_·4H_2_O | 94, 106, 148, 168, 211, 240, 286, 346, 382, 456, 480, 586, 607, 622, 659, 784, 990, 1071, 1096, 1146, 1590, 1629, 1679, 3334, 3376, 3438, 3533, 3593 | ^5^ |
| **FeSO_4_·7H_2_O** | 138, 185, 206, **241**, 264, **376**, 446, 465, 565, 619, 747, 976, 1075, 1102, 1138, 1625, 1648, 3371, 3436, 3506 | ^5^ |
| **FeSO_4_·7H_2_O** | 146, **191, 249, 377**, 450, 615, 980, 1106 | This work |
| NiO | 490 | ^6^ |
| α-Ni(OH)_2_ | 460, 711, 989, 1045, 3580, 3657 | ^7^ |
| β-Ni(OH)_2_ | 318, 449, 3580 | ^7^ |
| β-NiOOH | **480**, **560** | ^8^ |
| γ-NiOOH | **475**, **555** | ^8^ |
| α-FeOOH | 247, 299, 387, **475**, **549**, 689 | ^9^ |
| β-FeOOH | 302, 385, 490, 533, 612, 670, 708 | ^9^ |

Table S2. XPS fitting analysis for the O 1s in Ni_3_S_2_/Ni foam and Fe-Ni_3_S_2_/Ni foam.

|  | Atomic ratio of O | O 1s peak fitting ratio | | |
| --- | --- | --- | --- | --- |
|  |  | M-O | M-OH | adsorbed H_2_O |
| Ni_3_S_2_/Ni foam | 0.48 | 0.12 | 0.63 | 0.25 |
| Fe-Ni_3_S_2_/Ni foam | 0.60 | 0.47 | 0.21 | 0.32 |

Fig. S4. XPS signals of the (a) Ni 2p, (b) O 1s, (c) Fe 2p_3/2_ and (d) Fe 3p core level regions of Fe-Ni foam.

Fig. S5. Fe/Ni and S/Ni atomic ratios in (a) Ni_3_S_2_/Ni foam and (b) Fe-Ni foam at the surface (measured by XPS) and in the whole material (measured by ICP-AES).

Fig. S6 HRTEM images of nanostructures removed by sonication from Ni_3_S_2_/Ni foam.

Fig. S7. EDX elemental mapping images of nanostructures removed by sonication from Fe-Ni_3_S_2_/Ni foam.

Fig. S8. Fe/Ni and S/Ni atomic ratios in Fe-Ni_3_S_2_/Ni foam based on four different TEM elemental mapping areas (see Fig. S5).

Fig. S9. SEM images of (a, b) Ni foam and (c, d) Fe-Ni_3_S_2_/Ni foam.

Fig. S10. (a,b) SEM images of Fe-Ni foam. (c) TEM image of nanostructures removed by sonication from Fe-Ni foam.

Fig. S11. (a) The logarithm of iR-compensated linear sweep voltammetry (LSV) curves, (b) LSV curves without iR compensation and (c) uncompensated resistances (R) of Ni foam, Fe-Ni foam, Ni_3_S_2_/Ni foam and Fe-Ni_3_S_2_/Ni foam. (d) Nyquist plots of Ni foam and Fe-Ni_3_S_2_/Ni foam for OER, recorded at the overpotential of 400 mV. The signals at the high-frequency side are enlarged and shown in the inset. The measurements were conducted in 1.0 M KOH solution at 30 ℃.

Table S3. OER performance of state-of-the-art NiFe-based electrocatalysts investigated in 1.0 M KOH. The red numbers indicate the values of overpotential at 100 mA·cm^-2^ and/or the Tafel slope of the catalysts that are lower than those of our best catalyst (230 mV, 43 mV·dec^-1^).

| Catalysts | Substrate | Current density (mA·cm^-2^) | η (mV) | Tafel slope (mV·dec^-1^) | Ref. |
| --- | --- | --- | --- | --- | --- |
| Fe-Ni_3_S_2_ | Ni foam | 100 | 230 | 43 | This work |
| Pt-Ni(OH)_2_ | Ni mesh | 100 | 269 | 51 | ^10^ |
| Fe-NiCo_2_S_4_ | NiFe foam | 500 | 307 | 59 | ^11^ |
| Cu_2_S/CoFeCuOOH | Cu foam | 100 | 268 | 41 | ^12^ |
| FeNi_2_S_4_@Mo-doped Ni_3_S_2_ | Ni foam | 100 | 210 | 25 | ^13^ |
| NiCo-based phosphide | Ni foam | 50 | 446 | 147 | ^14^ |
| Fe-NiS_2_/Nitrogen-doped carbon nanotube | Carbon fiber cloth | 100 | 247 | 49 | ^15^ |
| NiCo-OH@Ni_x_Fe_y_O_4_ | Fe foam | 1000 | 275 | 23 | ^16^ |
| Cu_50_Ni_50_ nanoparticles | Glassy carbon plate | 10 | 319 | 64 | ^17^ |
| NiSe@NiFe-LDH | Ni foam | 100 | 232 | 58 | ^18^ |
| FeCo-Ni_3_S_4_ | Ni foam | 100 | 280 | 63 | ^19^ |
| FeOOH@β-Ni(Fe)OOH | Ni foam | 100 | 252 | 51 | ^20^ |
| Fe-Doped Ni_3_S_2_/FeS_2_ | Ni foam | 100 | 230 | 32 | ^21^ |
| NiCo-based hybrids | Ni foam | 100 | 331 | 82 | ^22^ |
| S-NiFe-LDH | MoNi foam | 50 | 280 | 40 | ^23^ |
| Activated Ni_9_S_8_/Ni_3_S_2_ N-doped carbon nanostructures | Carbon fiber | 20 | 310 | 46 | ^24^ |
| Ni-MOF@carbon nanotube | Glassy carbon | 10 | 244 | 47 | ^25^ |
| Fe-doped NiO coupled Ni cluster hollow nanotube arrays | Carbon fiber cloth | 10 | 245 | 43 | ^26^ |

Fig. S12 The (a) mass activity and (b) turnover frequency (TOF) of Ni foam, Ni_3_S_2_/Ni foam, Fe-Ni foam and Fe-Ni_3_S_2_/Ni foam. The mass activity is calculated based on the whole mass of the material (Table S4). The turnover frequency (TOF) is calculated by the following equation: ^27, 28^

$$TOF=\frac{jA}{\alpha Fn}$$

where *j* stands for the current density obtained from the LSV curves at a defined overpotential; *A* is the geometric surface area of the working electrode; *α* is the number of electrons involved in OER (4 electrons mol^–1^); *F* is the Faraday constant (96 485.3 C·mol^−1^); *n* represents the moles (mol) of metal atoms on the electrode, which is given by dividing the whole mass of the electrode (g) by the molar mass of metal (g·mol^-1^). Specifically, *n* = *n_foam_* (moles of Ni in the Ni foam) + *n_coating_* (moles of metal in the nanostructures coated on the Ni foam), where *n_coating_* is estimated using the formula:

$$n_{coating}=\text{ }\frac{wt\%_{Fe}\times m_{coating}}{\left(1-wt\%_{Ni}\right)\times M_{Fe}}$$

where *wt%_Fe_* and *wt%_Ni_* are the weight percent of Fe and Ni determined by XPS, respectively (Table S5); *m_coating_* is the mass of the coating on the Ni foam (Table S4); *M*_Fe_ is the molar mass of Fe. It should be noted that *m_coating_* does not include the mass of Ni in the nanostructures, as that Ni is originating from the Ni foam: this is the reason of the term (1 - *wt%_Ni_*) in the formula.

Table S4. Whole mass of Ni foam, Ni_3_S_2_/Ni foam, Fe-Ni foam and Fe-Ni_3_S_2_/Ni foam, and mass of the coating (estimated by subtracting the mass of the Ni foam from the mass of Ni_3_S_2_/Ni foam, Fe-Ni foam or Fe-Ni_3_S_2_/Ni foam, respectively).

|  | Ni foam | Ni_3_S_2_/Ni foam | Fe-Ni foam | Fe-Ni_3_S_2_/Ni foam |
| --- | --- | --- | --- | --- |
| whole mass [mg·cm^-2^] | 31 | 31.4 | 31.2 | 31.5 |
| mass of the coating [mg·cm^-2^] | 0 | 0.4 | 0.2 | 0.5 |

Table S5. Mass ratio of Ni_3_S_2_/Ni foam, Fe-Ni foam and Fe-Ni_3_S_2_/Ni foam based on XPS fitting data.

|  | Ni_3_S_2_/Ni foam | Fe-Ni foam | Fe-Ni_3_S_2_/Ni foam |
| --- | --- | --- | --- |
| Ni | 0.54 | 0.26 | 0.36 |
| Fe | 0.00 | 0.20 | 0.16 |
| S | 0.06 | 0.00 | 0.01 |
| O | 0.29 | 0.38 | 0.39 |
| C | 0.11 | 0.16 | 0.08 |

Fig. S13. CV curves at different scan rates (left) and corresponding C_dl_ (right): (a-b) Ni foam; (c-d) Fe‑Ni foam; (e-f) Ni_3_S_2_/Ni foam and (g-h) Fe-Ni_3_S_2_/Ni foam. Measurements were conducted in 1.0 M KOH solution at 30 ℃.

Fig. S14. Quasi-in-situ XPS signals of the Fe 2p core level region of Fe-Ni_3_S_2_/Ni foam corresponding to different potentials (vs. RHE) applied for 10 min. Electrochemical measurements were conducted in 1.0 M KOH solution at room temperature.

Fig. S15. In-situ Raman spectra of (a) Ni_3_S_2_/Ni foam and (b) Fe-Ni_3_S_2_/Ni foam recorded at OCP and after applying at 1.9 V vs. RHE for 30 s and 20 min. In-situ Raman spectra of (c) Ni_3_S_2_/Ni foam and (d) Fe-Ni_3_S_2_/Ni foam recorded at 1.2 V vs. RHE, before (yellow) and after (teal) exposing the electrocatalysts to a potential of 1.9 V vs. RHE.

Fig. S16. (a) Chronopotentiometric (CP) curves of Ni foam, Ni_3_S_2_/Ni foam and Fe-Ni_3_S_2_/NF at 500 mA·cm^-2^ for 100 h without iR compensation in 1.0 M KOH solution at 30 °C. (b) XRD patten and (c) Raman spectra of the dark powder discovered at the bottom of the electrolytic cell after a durability test at 500 mA·cm^-2^ for 100 h in 1.0 M KOH at 30 °C of Fe-Ni_3_S_2_/Ni foam.

Fig. S17. Raman spectra of Ni_3_S_2_/Ni foam and Fe-Ni_3_S_2_/Ni foam after conducting CP for 5 h.

Fig. S18. (a,b) SEM images of Fe-Ni_3_S_2_/Ni foam after 100 h durability test at 500 mA·cm^-2^ in 1.0 M KOH at 30 °C. (c) TEM and (d) HRTEM images of nanostructures removed by sonication from Fe-Ni_3_S_2_/Ni foam after 100 h durability test at 500 mA·cm^-2^ for 100 h in 1.0 M KOH at 30 °C.

Fig. S19. EDX elemental mapping images of nanostructures removed by sonication from Fe-Ni_3_S_2_/Ni foam after 100 h durability test at 500 mA·cm^-2^ for 100 h in 1.0 M KOH at 30 °C.

Fig. S20. Fe/Ni and S/Ni atomic ratios in Fe-Ni_3_S_2_/Ni foam after 100 h durability test at 500 mA·cm^-2^ for 100 h in 1.0 M KOH at 30 °C based on four different EDX elemental mapping areas (see Fig. S16).

Fig.S21. XPS signals of the (a) Ni 2p, (b) Fe 2p_3/2_, (c) Fe 3p, (d) S 2p and (d) O 1s core level regions of Fe-Ni_3_S_2_/Ni foam before and after 100 h durability test at 500 mA·cm^-2^ for 100 h in 1.0 M KOH at 30 °C.

Fig. S22. Fe/Ni and S/Ni atomic ratios in Fe-Ni_3_S_2_/Ni foam before and after 100 h OER durability test at 500 mA·cm^-2^ for 100 h in 1.0 M KOH at 30 °C based on (a) XPS (surface) and (b) ICP-AES (whole material).

## References

1. Cheng, Z.; Abernathy, H.; Liu, M., Raman spectroscopy of nickel sulfide Ni_3_S_2_. *J. Phys. Chem. C* **2007,** *111*, 17997-18000.

2. Sun, Y.; Wu, J.; Zhang, Z.; Liao, Q.; Zhang, S.; Wang, X.; Xie, Y.; Ma, K.; Kang, Z.; Zhang, Y., Phase reconfiguration of multivalent nickel sulfides in hydrogen evolution. *Energy Environ. Sci.* **2022,** *15*, 633-644.

3. Guillaume F.; Huang S.; Kenneth D. M. H.; Couzi M.; Talaga, D., Optical phonons in millerite (NiS) from single‐crystal polarized Raman spectroscopy. *J. Raman Spectrosc.* **2008,** *39*, 1419-1422.

4. Cui, R.; Li, D.; Fu, X.; Asif, M.; Pan, L., Growth of a carbon micro‐ and nanocoils mixture using NiSO_4_ as the catalyst precursor. *Chem. Vap. Deposition* **2015,** *21*, 78-83.

5. Chio, C. H.; Sharma, S. K.; Muenow, D. W., The hydrates and deuterates of ferrous sulfate (FeSO_4_): a Raman spectroscopic study. *J. Raman Spectrosc.* **2006,** *38*, 87-99.

6. Yeo, B. S.; Bell, A. T., In situ Raman study of nickel oxide and gold-dupported nickel oxide catalysts for the electrochemical evolution of oxygen. *J. Phys. Chem. C* **2012,** *116*, 8394-8400.

7. Hall, D. S.; Lockwood D. J.; Bock C.; MacDougall, B. R., Nickel hydroxides and related materials: a review of their structures, synthesis and properties. *Proc. R. Soc. A* **2015,** *471*, 20140792-20140857.

8. Lo, Y. L.; Hwang, B. J., In situ Raman studies on cathodically deposited nickel hydroxide films and electroless Ni-P electrodes in 1 M KOH solution. *Langmuir* **1998,** *14*, 944-950.

9. Mohammed, S.; Elnoor, M.; Hamad, I., The structural properties of iron oxides using Raman spectroscopy. *J. Sci. Eng. Res.* **2018,** *5*, 183-187.

10. Zhang, J.; Dang, J.; Zhu, X.; Ma, J.; Ouyang, M.; Yang, F., Ultra-low Pt-loaded catalyst based on nickel mesh for boosting alkaline water electrolysis. *Appl. Catal. B Environ.* **2023,** *325*, 122296-122306.

11. Wang, F.; Dong, Y.; Yu, C.; Dong, B.; Zhang, X.; Fan, R.; Xie, J.; Zhou, Y.; Chai, Y., Trojan strategy assisted phase-pure Fe-NiCo_2_S_4_ for industrial anion-exchange membrane water electrolyzer. *Appl. Catal. B Environ.* **2023,** *331*, 122660-122670.

12. Wang, C.; Zhai, P.; Xia, M.; Liu, W.; Gao, J.; Sun, L.; Hou, J., Identification of the origin for reconstructed active sites on oxyhydroxide for oxygen evolution reaction. *Adv. Mater.* **2023,** *35*, 2209307-2209320.

13. Phadikar, U.; Das, S.; Bolar, S.; Kundu, A.; Kolya, H.; Kang, C. W.; Murmu, N. C.; Kuila, T., Sulfur scrambling assisted in-situ growth of 3D - hierarchical FeNi_2_S_4_@Mo-doped Ni_3_S_2_/NF nanosheet arrays: A stellar performer towards alkaline water electrolysis. *J. Power Sources* **2023,** *576*, 233244-233255.

14. Park, D. H.; Kim, M. H.; Kim, M.; Byeon, J. H.; Jang, J. S.; Kim, J. H.; Lim, D. M.; Park, S. H.; Gu, Y. H.; Kim, J.; Park, K. W., Spherical nickel doped cobalt phosphide as an anode catalyst for oxygen evolution reaction in alkaline media: From catalysis to system. *Appl. Catal. B Environ.* **2023,** *327*, 122444-122455.

15. Liu, X.; Zhao, X.; Cao, S.; Xu, M.; Wang, Y.; Xue, W.; Li, J., Local hydroxyl enhancement design of NiFe sulfide electrocatalyst toward efficient oxygen evolution reaction. *Appl. Catal. B Environ.* **2023,** *331*, 122715-122725.

16. Li, Z.; Zhang, X.; Zhang, Z.; Chen, P.; Zhang, Y.; Dong, X., Dual-metal hydroxide@oxide heterojunction catalyst constructed via corrosion engineering for large-current oxygen evolution reaction. *Appl. Catal. B Environ.* **2023,** *325*, 122311-122321.

17. Gioria, E.; Li, S.; Mazheika, A.; d’Alnoncourt, R. N.; Thomas, A.; Rosowski, F., CuNi nanoalloys with tunable composition and oxygen defects for the enhancement of the oxygen evolution reaction. *Angew. Chem. Int. Ed.* **2023,** *62*, 202217888-202217898.

18. Bao, W.; Yang, C.; Ai, T.; Zhang, J.; Zhou, L.; Li, Y.; Wei, X.; Zou, X.; Wang, Y., Modulating interfacial charge distribution of NiSe nanoarrays with NiFe-LDH nanosheets for boosting oxygen evolution reaction. *Fuel* **2023,** *332*, 126227-126235.

19. Mao, X.; Liu, Y.; Chen, Z.; Fan, Y.; Shen, P., Fe and Co dual-doped Ni_3_S_4_ nanosheet with enriched high-valence Ni sites for efficient oxygen evolution reaction. *Chem. Eng. J.* **2022,** *427*, 130742-130751.

20. Li, Y.; Wu, Y.; Yuan, M.; Hao, H.; Lv, Z.; Xu, L.; Wei, B., Operando spectroscopies unveil interfacial FeOOH induced highly reactive β-Ni(Fe)OOH for efficient oxygen evolution. *Appl. Catal. B Environ.* **2022,** *318*, 121825-121836.

21. Gultom N. S.; Li C. H.; Kuo, D. H.; Abdullah, H., Single-step synthesis of Fe-doped Ni_3_S_2_/FeS_2_ nanocomposites for highly efficient oxygen evolution reaction. *ACS Appl. Mater. Interfaces* **2022,** *14* , 39917-39926.

22. Chen, M.; Liu, D.; Feng, J.; Zhou, P.; Qiao, L.; Feng, W.; Chen, Y.; Ng, K. W.; Wang, S.; Ip, W. F.; Pan, H., In-situ generation of Ni-CoOOH through deep reconstruction for durable alkaline water electrolysis. *Chem. Eng. J.* **2022,** *443*, 136432-136440.

23. Zhou, Y.; Yu, W.; Cao, Y.; Zhao, J.; Dong, B.; Ma, Y.; Wang, F.; Fan, R.; Zhou, Y.; Chai, Y., S-doped nickel-iron hydroxides synthesized by room-temperature electrochemical activation for efficient oxygen evolution. *Appl. Catal. B Environ.* **2021,** *292*, 120150-120158.

24. Thangasamy, P.; Nam, S.; Randriamahazaka, S. O. H.; Oh, II-K., Boosting oxygen evolution reaction on metallocene-based transition metal sulfides integrated with N-doped carbon nanostructures. *ChemSusChem* **2021,** *14* , 5004-5020.

25. Srinivas, K.; Chen, Y.; Wang, X.; Wang, B.; Karpuraranjith, M.; Wang, W.; Su, Z.; Zhang, W.; Yang, D., Constructing Ni/NiS heteronanoparticle-embedded metal–organic framework-derived nanosheets for enhanced water-splitting catalysis. *ACS Sustainable Chem. Eng.* **2021,** *9*, 1920-1931.

26. Lei, Y.; Xu, T.; Ye, S.; Zheng, L.; Liao, P.; Xiong, W.; Hu, J.; Wang, Y.; Wang, J.; Ren, X.; He, C.; Zhang, Q.; Liu, J.; Sun, X., Engineering defect-rich Fe-doped NiO coupled Ni cluster nanotube arrays with excellent oxygen evolution activity. *Appl. Catal. B Environ.* **2021,** *285*, 119809-119819.

27. Li, L.; Wang, P.; Shao, Q.; Huang, X., Metallic nanostructures with low dimensionality for electrochemical water splitting. *Chem. Soc. Rev.* **2020,** *49*, 3072-3106.

28. Guo, Y.; Park, T.; Yi, J. W.; Henzie, J.; Kim, J.; Wang, Z.; Jiang, B.; Bando, Y.; Sugahara, Y.; Tang, J.; Yamauchi, Y., Nanoarchitectonics for transition-metal-sulfide-based electrocatalysts for water splitting. *Adv. Mater.* **2019,** *31*, 1807134-1807168.
